# *Ranbp1* modulates morphogenesis of the craniofacial midline in mouse models of 22q11.2 deletion syndrome

**DOI:** 10.1093/hmg/ddad030

**Published:** 2023-02-15

**Authors:** Elizabeth M Paronett, Corey A Bryan, Megan E Maynard, Julia A Goroff, Daniel W Meechan, Anthony-Samuel LaMantia, Thomas M Maynard

**Affiliations:** Department of Anatomy and Cell Biology, The George Washington School of Medicine and Health Sciences, Washington, DC 20037, USA; Department of Anatomy and Cell Biology, The George Washington School of Medicine and Health Sciences, Washington, DC 20037, USA; Center for Neurobiology Research, The Fralin Biomedical Research Institute at Virginia Tech Carilion School of Medicine, Roanoke, VA 24014, USA; Department of Anatomy and Cell Biology, The George Washington School of Medicine and Health Sciences, Washington, DC 20037, USA; Center for Neurobiology Research, The Fralin Biomedical Research Institute at Virginia Tech Carilion School of Medicine, Roanoke, VA 24014, USA; Center for Neurobiology Research, The Fralin Biomedical Research Institute at Virginia Tech Carilion School of Medicine, Roanoke, VA 24014, USA; Department of Biological Sciences, Virginia Tech, Blacksburg, VA 24060, USA; Center for Neurobiology Research, The Fralin Biomedical Research Institute at Virginia Tech Carilion School of Medicine, Roanoke, VA 24014, USA

## Abstract

Facial dysmorphology is a hallmark of 22q11.2 deletion syndrome (22q11DS). Nearly all affected individuals have facial features characteristic of the syndrome: a vertically long face with broad nasal bridge, narrow palpebral fissures and mild micrognathia, sometimes accompanied by facial skeletal and oropharyngeal anomalies. Despite the frequency of craniofacial dysmorphology due to 22q11.2 deletion, there is still incomplete understanding of the contribution of individual 22q11 genes to craniofacial and oropharyngeal development. We asked whether homozygous or heterozygous loss of function of single 22q11 genes compromises craniofacial and/or oropharyngeal morphogenesis related to these 22q11DS phenotypes. We found that *Ranbp1*, a 22q11DS gene that mediates nucleocytoplasmic protein trafficking, is a dosage-dependent modulator of craniofacial development. *Ranbp1^−/−^* embryos have variably penetrant facial phenotypes, including altered facial morphology and cleft palate. This 22q11DS-related dysmorphology is particularly evident in the midline of the facial skeleton, as evidenced by a robustly quantifiable dysmorphology of the vomer, an unpaired facial midline bone. 22q11DS-related oropharyngeal phenotypes reflect *Ranbp1* function in both the cranial neural crest and cranial ectoderm based upon tissue-selective *Ranbp1* deletion. Analyses of genetic interaction show that *Ranbp1* mutation disrupts BMP signaling-dependent midline gene expression and BMP-mediated craniofacial and cranial skeletal morphogenesis. Finally, midline defects that parallel those in *Ranbp1* mutant mice are observed at similar frequencies in the *LgDel* 22q112DS mouse model. Apparently, *Ranbp1* is a modulator of craniofacial development, and in the context of broader 22q11 deletion, *Ranbp1* mutant phenotypes mirror key aspects of 22q11DS midline facial anomalies.

## Introduction

Orofacial anomalies are a significant clinical feature of 22q11.2 deletion syndrome (22q11DS), a heterozygous microdeletion disorder affecting 1 in 4000 individuals (1). In one of the earliest descriptions of what was then named velocardiofacial syndrome, Shprintzen *et al*. (2) noted that ‘the most striking feature of these patients was the similar facies’, characterized by features including a broad nasal bridge, narrow palpebral fissures, retruded mandible and a long face. More recent quantitative 3d morphometric analysis has found evidence for narrowing of the lower face and the nasal prominences, suggesting that the facial midline may be compromised (3). In addition, oropharyngeal structure and function is often disrupted in individuals with 22q11DS. Overt cleft palate is frequent (~10%); however, a majority (~65%) of individuals with 22q11DS have submucosal cleft palate, bifid uvula and/or velopharyngeal dysfunction (4). These latter anomalies can impair suckling and swallowing during early life (5,6) as well as solid food ingestion, swallowing, and speech during maturation and adulthood (7). There is little insight into how individual 22q11DS candidate genes contribute to craniofacial dysmorphogenesis or oropharyngeal pathogenesis (8) that underly these clinical complications in 22q11DS. We asked whether *Ranbp1*, a 22q11 gene expressed in the neural tube, neural crest, cranial ectoderm and oropharyngeal primordia (9), may be a key genetic modulator of craniofacial development, including establishment of midline craniofacial structures in the context of 22q11DS.

Previous studies of the contribution of individual 22q11DS-deleted genes to oropharyngeal and craniofacial dysmorphology have focused on *Tbx1*, a transcription factor that regulates cardiac and cranial mesoderm and endoderm. There is clear evidence for *TBX1* as a causal gene for human cardiac anomalies (10,11), but it appears unlikely that *TBX1* heterozygosity is primarily responsible for facial dysmorphology including cleft palate in 22q11DS (12). Homozygous null mutations of *Tbx1*^−/−^ in mouse embryos results in a highly penetrant cleft palate defect (13); however, heterozygous *Tbx1* deletion does not lead to recognizable craniofacial phenotypes (14,15). In contrast, we have reported partially penetrant palatal closure defects (16) and cranial skeletal anomalies (17) in the *LgDel* mouse model of 22q11DS, which carries a heterozygous deletion orthologous to the minimal critical deletion associated with 22q11DS in humans, resulting in diminished dosage of *Tbx1* and 27 other murine 22q11 orthologues (15). The absence of identifiable craniofacial dysmorphology in *Tbx1*^+/−^ mice and the presence of craniofacial dysmorphology in models of broader 22q11 gene deletion implicates additional 22q11 genes in the disruption of craniofacial development by 22q11DS. We have shown previously that multigenic 22q11 deletion has substantially different consequences for craniofacial, cranial nerve and cardiovascular morphogenetic signaling than heterozygous *Tbx1* mutation (16,18,19). Thus, it is likely that craniofacial morphogenesis is influenced by additional heterozygously deleted 22q11 genes.

We focused on *Ranbp1* as a potential modulator of multiple 22q11DS morphogenetic phenotypes based upon its enhanced expression in neural crest and craniofacial primordia (9), its capacity to disrupt hindbrain patterning and cranial nerve differentiation (19), as well as its identity as a microcephaly gene based upon selectively disrupted neurogenesis in the cerebral cortex in *Ranbp1*^−/−^ embryos (20). In addition, its established cellular function as a binding partner and regulator of the small G protein RAN, responsible for modulating nucleocytoplasmic shuttling (21) via binding to the Ran/Exportin complex (22), suggests that Ranbp1 may facilitate morphogenetic signaling that relies upon efficient trafficking of transcriptional regulators that transit between the nucleus and cytoplasm. We used constitutive and conditional *Ranbp1* mutants as well as additional 22q11DS murine models to assess the contribution of *Ranbp1* function to specific aspects of facial, palatal and cranial skeletal morphogenesis. We found that *Ranbp1* mutation disrupts midline facial and palatal morphogenesis independently as well as in the context of broader 22q11 deletion. Our results establish *Ranbp1* as a regulator of multiple midline facial and oropharyngeal dysmorphologies that are also characteristic of 22q11DS.

## Results

### *Ranbp1* loss-of-function leads to facial anomalies and palate defects

**Figure 1:**
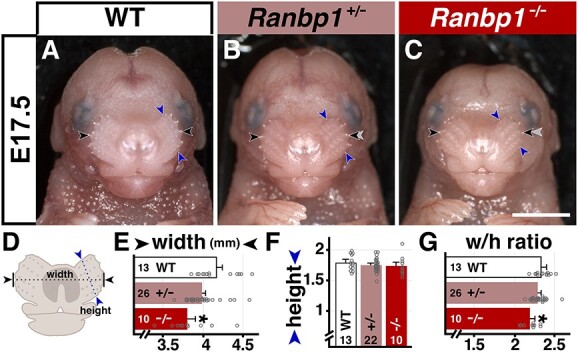
Gross phenotype of non-exencephalic *Ranbp1* homozygotes. (**A**–**C**) WT, *Ranbp1*^+/−^ and ^−/−^ embryos at E17.5 in frontal view. (**D**–**G**) Measurement of facial (whisker pad) width and height from frontal view of E17.5 embryos. (D) Illustration of measurements. Black and blue arrows in A–D denote locations of width and height measurements. Gray arrows in B and C mark the width noted in A to facilitate visual comparisons. (E) Width in null embryos differs from WT (*P* = 0.003 by one-way ANOVA with Šídák's MCT). (F) Height of null embryos does not significantly differ from WT (*P* > 0.7). (G) Width/height ratio supports hypothesis that width is more significantly impacted than height; w/h in null embryos differs significantly from WT (*P* = 0.001). Scale bars = 0.25 cm for A–C.

We first quantified craniofacial dysmorphology in embryos constitutively lacking functional *Ranbp1*, using a gene-trap mutation previously described as a robust loss-of-function allele (20). *Ranbp1* null mutants do not survive after birth. Roughly half of *Ranbp1*^−/−^ embryos have grossly observable craniofacial anomalies by late gestation. The other half of late gestation *Ranbp1*^−/−^ embryos are exencephalic, a phenotype that is not representative of the 22q11DS clinical population. Thus, we focused our initial analysis on the non-execephalic cohort of mouse embryos, since phenotypes in this group are likely more relevant to those that arise from *Ranbp1* diminished dosage in the context of 22q11DS. We first assessed facial morphology in late gestation *Ranbp1*^−/−^ embryos. At E17.5, facial proportions in *Ranbp1*^−/−^ embryos are altered, especially in medio-lateral dimensions (Fig. 1A–C). A quantitative assessment confirms that the width of the *Ranbp1*^−/−^ face (width across whisker pads; Fig. 1E) is diminished significantly from that in *Ranbp1*^+/+^ (WT) or *Ranbp1*^+/−^ embryos (91%, of WT; *P* = 0.003, *P* = 0.008, respectively, one-way analysis of variance [ANOVA] with Šídák's multiple comparisons test [MCT]). In contrast, dorsal–ventral (D-V) height does not differ significantly (whisker pad height; Fig. 1F). *Ranbp1*^−/−^ late gestation embryos are somewhat smaller than their WT littermates, including a smaller cranial size associated with microcephaly (20); however, the change in facial width does not seem to simply reflect reduced embryonic size. Instead, there is a proportional change in width versus height in the faces of E17.5 *Ranbp1*^−/−^ embryos that indicates a selective narrowing of facial structures in the M-L but not D-V dimension (Fig. 1G). This is further supported by similar findings using measures of additional facial landmarks, including lip and mouth width versus jaw height (Supplementary Material, Fig. S1).

**Figure 2:**
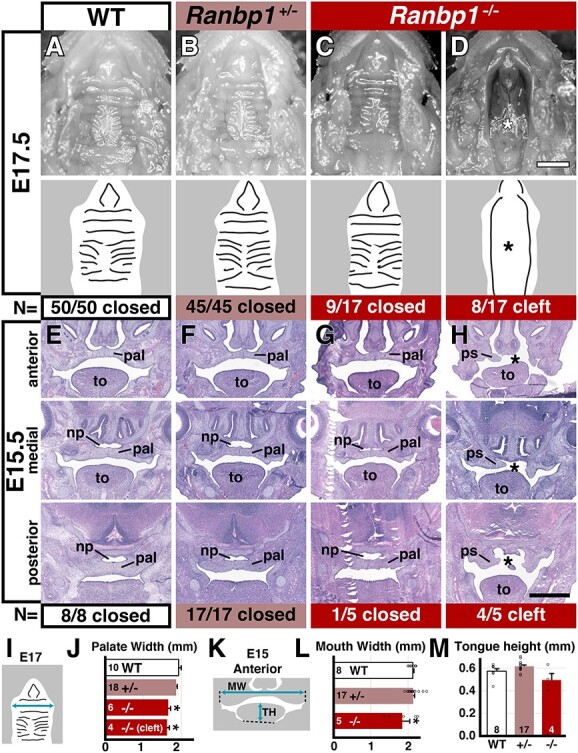
Palate defects in *Ranbp1* null embryos. (**A**–**D**) Dissected palate/upper jaw at E17.5 for in WT (A), heterozygous (B) and non-exencephalic null embryos with a normal/closed (C) or cleft palate (D). Line drawing below each image illustrates palatal features. Asterisk marks overt cleft; dashed line indicates arch. (**E**–**H**) Hematoxylin and eosin-stained sections of E15.5 embryos, from three planes of section: anterior to the eye, medial (at the level of the eye) and posterior to the eye, detailing above phenotypes. Abbreviations: pa, palatal shelf; to, tongue; np, nasopharynx. (**I**) Measurement of palatal width in dissected preparations at level of second ruga; (**J**) palate of cleft and non-cleft null embryos are narrower than WT (*P* < 0.002 for both, one-way ANOVA with Šídák's MCT). (**K**) Measurement in anterior E15.5 sections. (**L**) Mouth is narrower in *Ranbp1*^−/−^ (*P* = 0.04 versus WT). (**M**) Tongue height does not differ between *Ranbp1*^−/−^ and WT (*P* = 0.14 versus WT). Scale bar = 1 mm for A–D, 0.5 mm for E–H.

In 22q11DS, outwardly visible facial dysmorphology is often accompanied by oropharyngeal dysmorphology. Approximately 10% of individuals with 22q11DS have overt cleft palate, usually without a cleft lip (4). To assess whether *Ranbp1* contributes to typical palate development, and if loss-of-function disrupts this key target of 22q11DS pathogenesis, we assessed the gross morphology of the developing palate in a cohort of E17.5 WT, *Ranbp1*^+/−^ and non-exencephalic *Ranbp1*^−/−^ embryos (Fig. 2A–D), and confirmed these findings by scanning electron microscopy of examples of key phenotypes (Supplementary Material, Fig. S2). In WT embryos, both primary (the anterior/medial portion of the palate, adjacent to the upper lip and incisors) and secondary (posterior) palates are closed and apparently developing normally (Fig. 2A; Supplementary Material, Fig. S2A). In contrast, *Ranbp1*^−/−^ embryos have a partially penetrant clefting phenotype. Roughly half have an overt cleft palate (8/17 embryos; Fig. 2C and D). In *Ranbp1*^−/−^ embryos with a cleft (Fig. 2D, Supplementary Material, Fig. S2B), overt clefting spans the entire secondary palate, but spares the primary (anterior-most) palate, consistent with the lack of a cleft lip phenotype. To further characterize this clefting phenotype, we complemented these gross analyses with a histological analysis of sections at three anterior/posterior levels at E15.5 (Fig. 2E–H). The palatal shelves of WT and *Ranbp1*^+/−^ embryos are closed and fused by E15.5 to produce an intact secondary palate (Fig. 2E and F). However, in *Ranbp1*^−/−^ embryos, most (4/5) had palatal shelves that failed to properly close (Fig. 2H), a common precursor to a cleft palate phenotype. Clefting does not occur in heterozygous *Ranbp1* embryos (Fig. 2B and F): 62/62 *Ranbp1*^+/−^ embryos have intact, closed palates when observed between E15.5 and 17.5. Nevertheless, these embryos still have quantifiable midline cranial skeletal defects (see below). Similar to the apparent relationship between *Ranbp1*^−/−^ and *Ranbp1*^−/+^ mutations for palate morphogenesis, *Tbx1^−/−^* mutations are associated with palatal clefting; however, *Tbx1*^+/−^ mutations are not. Thus, to determine whether overt cleft palate in the context of broader 22q11 deletion reflects interactions between heterozygous loss-of-function of *Tbx1* and *Ranbp1* we analyzed palate morphogenesis in *Tbx1*^+/−^;*Ranbp*^+/−^ embryos. We found no evidence for interactions between the two genes, as 0/15 *Tbx1*^+/−^;*Ranbp*^+/−^ embryos show clefting between E15.5 and 17.5.

The dissected palates of *Ranbp1*^−/−^ embryos appeared slightly narrower than their WT littermates (e.g. Fig. 2A versus C/D). We quantified this by assessing the width of the mouth in two ways: first, by directly measuring palate dimensions in E17.5 dissected palates (Fig. 2I and J), or second, by measuring the defined oral opening in coronal sections of E15.5 embryos (Fig. 2 K and L). In E17.5 dissected preparations, the mouth (across the palate at the level of the second palatal ruga, Fig. 2I) is narrower in both non-cleft and cleft embryos (~85% of WT, *P* < 0.002 for both, one-way ANOVA with Šídák's MCT; Fig. 2J). Similarly, the width of the anterior mouth in E15.5 sections (Fig. 2K) is also narrower in *Ranbp1*^−/−^ embryos (~86% of WT, *P* = 0.04 as above; Fig. 2K and L). In contrast, tongue height in *Ranbp1*^−/−^ embryos does not differ from that in WT littermates (Fig. 2M), suggesting that shelf fusion and subsequent palate closure in *Ranbp1*^−/−^ embryos is not likely impaired by an enlarged tongue as in some previously reported mutations (23,24).

These changes in facial morphology also do not appear to be secondary to a decline in embryonic viability: although cardiac anomalies are frequent in human 22q11DS and *LgDel* mice (4,15), *Ranbp1*^−/−^ embryos appear as well perfused as their WT littermates, and a preliminary examination of four *Ranbp1*^−/−^ embryos by intracardiac dye injection revealed intact aortic arches and appropriately filled left and right carotid arteries (data not shown). Thus, the most severe cardiac anomalies associated with 22q11DS (interrupted aortic arch and/or tetralogy of Fallot) are not likely a significant feature of *Ranbp1*^−/−^ embryos. Finally, there was no noticeable limb, digit or tail dysmorphology in the late gestation *Ranbp1*^−/−^ embryos we analyzed. Accordingly, craniofacial and cranioskeletal anomalies emerge as central, specific, phenotypes due to *Ranbp1* mutation for further cellular, genetic and mechanistic analysis of the contribution of *Ranbp1* function.

### *Ranbp1* mutation does not significantly impact palatal shelf proliferation or apoptosis

**Figure 3:**
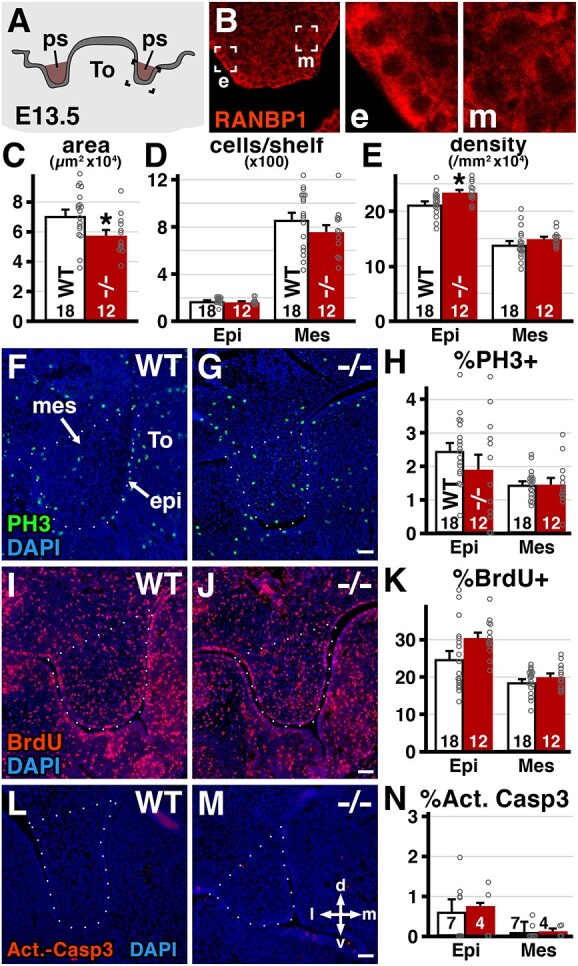
Quantification of growth and cell proliferation of palatal shelves. (**A**, **B**) Expression of RANBP1 protein in palatal shelves. (A) Location of imaged area from sectioned E13.5 embryos at the level of the eye. (B) Confocal image of palatal shelf labeled with anti-RANBP1 antibody showing expression in both epithelium (e) and mesenchyme (m). (C–E) Measurement of palatal shelves from cryosections. (**C**) Shelves are smaller in nulls (*P* = 0.01 by *t*-test). (**D**) Total number of cells per shelf is similar in nulls versus WT (epi: *P* > 0.9, mes: *P* > 0.16 by one-way ANOVA with Šídák's MCT) (**E**) Cell density is slightly higher in epithelium (*P* = 0.028) but not mesenchyme (*P* > 0.37). (**F**–**H**) Proliferation as quantified by the M-phase marker Phospho-histone-H3 (PH3). PH3+ cells are not significantly different in either epithelium or mesenchyme at E13.5 (epi: *P* > 0.53, mes: *P* > 0.99). (**I**–**K**) Proliferation is not significantly different as measured by (BrdU) incorporation following a 1 h exposure (epi: *P* > 0.13, mes: *P* > 0.83). (**L**–**N**) Apoptotic cell death as measured by activated Caspase 3 staining; few cells are present and they are not significantly different in WT versus *Ranbp1*^−/−^ (epi: *P* > 0.9, mes: *P* > 0.9). Number of shelves quantified noted on each graph. Borders of palatal shelf indicated in F–G, I–J and K–L by dotted lines; d/v and l/m arrows provide orientation to dorsal/ventral and lateral/medial axis. Scale bars = 50 μm.

In multiple genetic models, altered cell proliferation or cell death in the palatal shelf epithelium and/or mesenchyme shortly after these structures form (around E12–13) prefigures the failure of palatal shelves to grow, elevate and fuse to form an intact secondary palate (25–29). We therefore asked whether there were detectable differences in palatal shelf cell proliferation in WT versus *Ranbp1*^−/−^ embryos at E13.5, when palatal shelves are first beginning to grow out of the dorsolateral oral cavity (Fig. 3A). We first assessed expression of *Ranbp1* in the palatal shelf epithelium and mesenchyme to determine whether the protein is available in the WT tissues to potentially influence cellular function, and absent in the *Ranbp1*^−/−^ mutant (Fig. 3B), RANBP1 protein is robustly expressed in both tissues, although the relative expression level appears higher in oral epithelium at this stage. We next analyzed the size of the palatal shelves as well as cell density in the shelf epithelium and mesenchyme of WT and *Ranbp1*^−/−^ E13.5 embryos. Palatal shelves in *Ranbp1*^−/−^ embryos are slightly smaller in cross-sectional area (81% of WT; *P* = 0.03 by *t*-test; Fig. 3C), although the total number of cells present in each shelf is similar (Fig. 3D; epi: *P* > 0.9, mes: *P* > 0.16 by one-way ANOVA). Accordingly, we found a significant increase in cell density in the palatal shelf epithelium in *Ranbp1*^−/−^ E13.5 embryos (Fig. 3E; *P* = 0.028) but not mesenchyme (*P* > 0.37). We next assessed proliferation using two independent methods: labeling of cells entering M-phase by phosphorylated histone 3 protein expression (PH3+, Fig. 3F–H), or cells in S-phase based upon acute 5-bromo-2′-deoxyuridine (BrdU) incorporation (Fig. 3I–K). There were no statistically significant changes in cell proliferation in the E13.5 *Ranbp1*^−/−^ palatal shelf epithelium or mesenchyme measured by either method (PH3 epi: *P* > 0.53, mes: *P* > 0.99 by one-way ANOVA; Fig. 3F–H; BrdU epi: *P* > 0.13, mes: *P* > 0.83; Fig. 3). Finally, there is little apoptotic death in either WT or *Ranbp1*^−/−^ shelves at this time in midgestation (Fig. 3K–M). Activated caspase-3 labeled cells are rare in both the epithelium and mesenchyme (*P* > 0.9 versus WT for epi and mes). Thus, it appears that palatal clefting due to *Ranbp1* loss of function is unlikely to be due to disrupted proliferation or increased apoptosis during this well-established period of initial palatal shelf morphogenesis and maturation. The increased cell density in the palatal epithelium suggests that altered morphogenetic processes constrain epithelial growth without a concomitant change in cell size or number.

### *Ranbp1* loss of function disrupts midline cranial skeletal morphogenesis

**Figure 4:**
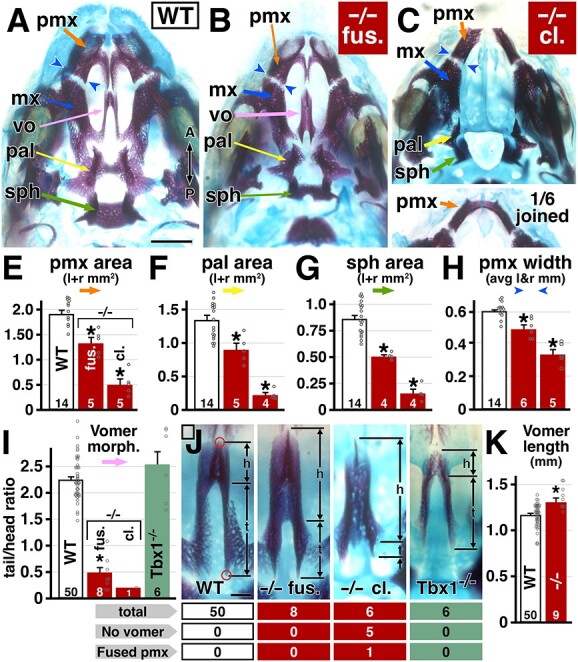
Cranial bone phenotype in *Ranbp1* mutant embryos at E17.5. (**A**–**C**) Alcian blue/alizarin red staining of upper jaw and palate for WT (A), and null mutants with fused/closed palate (fus, B) and null mutants with cleft palate (cl., C) phenotypes. Key structures are noted: premaxilla (pmx), maxilla (mx), vomer (vo), palatine (pal) and sphenoid (sph). (**E**–**H**) Areas were measured for premaxilla (E), palatine (F), basisphenoid (G) and width of premaxilla (H) measured at maxilla-premaxilla suture (purple arrows in Fig. 4A–C). Asterisks for (E–H) denote difference from WT by one-way ANOVA with Šídák's MCT (*P* < 0.01). (**I**–**K**) Quantification of vomer morphology. Vomers were photographed in a slightly oblique view (tilted laterally) to allow clear observation of anterior and posterior ends (red circles in J), then the extent of the ‘head’ (perpendicular plate) and ‘tail’ (alar) domains were measured; left and right-side measures are averaged for each specimen. Tail/head ratio was calculated for each specimen (I). Asterisks denote statistical difference from WT (*P* < 0.01 by one-way ANOVA of all constitutive alleles in study, with Šídák's MCT). (J) Example vomers for each quantified genotype. (K) Total vomer length of all WT embryos is slightly shorter than length of all *Ranbp1*^−/−^ embryos (*P* = 0.001 by *t*-test). Scale bar = 1 mm for A–C, 0.2 mm for J. N for each subclass is listed below, along with fraction that did not have a detectable vomer.

The dysmorphology of the soft tissues of the palate in *Ranbp1*^−/−^ embryos suggests that there may be accompanying disruption of craniofacial skeletal elements within and adjacent to the palate. Thus, we measured sizes of palate-related and adjacent cranial bones from WT and *Ranbp1*^−/−^ wholemount cranial skeletons stained with Alizarin Red to label bone, and Alcian Blue to label cartilage. There is substantial cranial skeletal dysmorphology in *Ranbp1*^−/−^ embryos at E17.5 (Fig. 4A–C). Most, if not all, of the facial bones appear smaller in null embryos. For the subset of null embryos without overt clefting (−/− fus.; Fig. 4B), areal measurements confirm that the premaxilla (pmx; Fig. 4E; 69% of WT; *P* < 0.001 by one-way ANOVA with Šídák's MCT), palatine (pl; Fig. 4F; 66% of WT; *P* = 0.003) and sphenoid (Fig. 4G; 59% of WT; *P* = 0.002) are all significantly smaller. Similarly, the premaxilla (mx) is thinner (Fig. 4H; 81% of WT, measured at the maxilla/premaxilla suture; *P* < 0.001). In null embryos with an overt cleft, the dysmorphology is even more pronounced: the premaxilla (pmx; Fig. 4E; 26% of WT; *P* < 0.001), palatine (pl; Fig. 4F; 16% of WT; *P* < 0.001) and sphenoid (Fig. 4G; 18% of WT; *P* < 0.001) are significantly smaller, and the premaxilla (mx) is thinner (Fig. 4H; 59% of WT; *P* < 0.001). In one null embryo (1/6 −/− with an overt cleft palate), the premaxilla appears as a singular bone joined at the anterior midline (Fig. 4C, lower).

The appearance of a fused premaxilla along with the narrow facial features and palatal anomalies of *Ranbp1*^−/−^ embryos suggests that the organization of the craniofacial midline skeleton may be disrupted. Thus, we quantified the morphology of a consistently identifiable midline bone, the vomer, the only unpaired midline facial bone at this stage, which appears as an ‘upside-down Y’ shape dorsal to the maxilla and palate bones (Fig. 4A). In every *Ranbp1*^−/−^ E17.5 embryo we analyzed (*n* = 19/19), the vomer is highly dysmorphic (see Fig. 4B) or absent (see Fig. 4C). To determine whether these changes reflect targeted dysmorphogenesis or size change proportionate to a broader failure of *Ranbp1*^−/−^ fetal growth, we analyzed non-exencephalic *Ranbp1*^−/−^ and WT vomers morphometrically. In the subset of *Ranbp1*^−/−^ embryos in which a vomer remains, there is an apparent proportional increase in the size of the anterior ‘head’ domain (the perpendicular plate), at the expense of the paired posterior ‘tail’ domain (the alae). To quantify this dysmorphology, we developed a strategy to measure consistently the size and proportions of the vomer (Fig 4I and J). We imaged the vomer in cleared preparations, tilting slightly laterally to identify the anterior and posterior termini of the bone (marked with red circles in Fig. 4J). In these images, we then measured the ‘tail’ and ‘head’ of each bone for both left and right sides to calculate an average ‘tail/head’ ratio (Fig. 4I) for each specimen. This ratiometric scoring efficiently evaluates morphology independent of differences in the absolute size of the specimen or embryo. When the vomer is present in *Ranbp1*^−/−^ embryos, it is highly dysmorphic and clearly distinguishable from those in WT littermates. Our morphometric analysis confirms the robustness of this apparent distinction. In WT embryos, the vomer tail is significantly longer than the head (t/h ratio average = 2.25 ± 0.05), but in *Ranbp1*^−/−^ embryos without cleft palate the proportions are inverted (Fig. 4I; t/h ratio = 0.49 ± 0.09; *P* = 0.0001 versus WT by one-way ANOVA with Šídák's test; see also Supplementary Material, Fig. S3A), and the ratio is even more dramatic in the single non-exencephalic null embryo with a cleft palate that had a visible vomer (t/h ratio = 0.20). Finally, the vomer in non-exencephalic null embryos is slightly, but significantly, longer than it is in WT (*Ranbp1*^−/−^ vomer length is 116% of WT, *P* = 0.001 by *t*-test; Fig. 4K; see also Supplementary Material, Fig. S3B), reinforcing the conclusion that this morphological anomaly is not due to the slightly smaller size of *Ranbp1*^−/−^ embryos.

We next asked whether vomer dysmorphology is a primary feature of *Ranbp1* loss of function, or also seen within the spectrum of oropharyngeal dysmorphology, including secondary cleft palate, mediated by diminished dosage of *Tbx1*, another 22q11 deleted gene associated with oropharyngeal dysmorphology. The vomer is present in *Tbx1*^−/−^ embryos (*n* = 6/6) despite each having an overt secondary palate cleft. There is general hypotrophy in the *Tbx1*^−/−^ cranial skeleton (Fig. 4J, Supplementary Material, Fig. S4), but *Tbx1*^−/−^ vomer morphology remains morphologically comparable to WT (Fig. 4I–J; t/h ratio for *Tbx1*^−/−^ is ~112% of WT; *P* > 0.4; see also Supplementary Material, Fig. S4). Thus, the midline cranial skeletal disruption in *Ranbp1*^−/−^ embryos is independent of secondary palate clefting, and distinct from malformations due to *Tbx1* loss of function. Apparently, *Ranbp1* disrupts distinct dimensions of craniofacial skeletal as well as facial and oropharyngeal morphogenesis, and may therefore make a distinct contribution to phenotypic variation in midline craniofacial dysmorphology and palatal anomalies associated with broader 22q11 deletion.

### Mesenchymal/epithelial interaction and palate/midline oropharyngeal skeletal development

**Figure 5:**
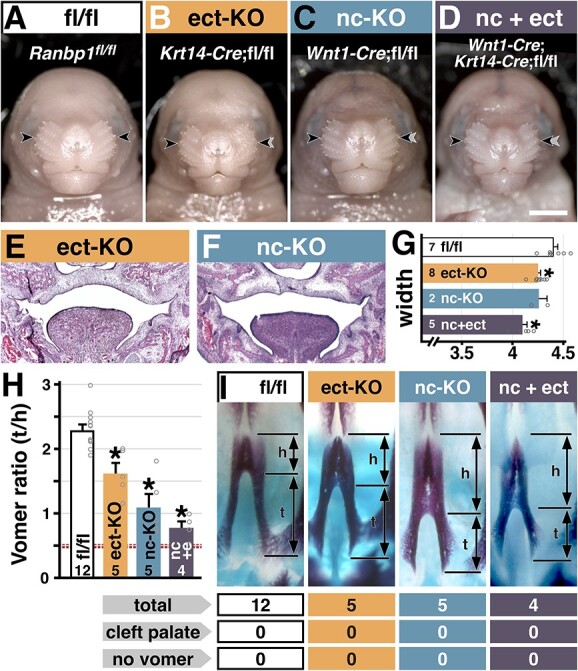
Conditional cre-lox ablation of *Ranbp1* from neural crest and oral epithelial/ectodermal compartments, using *Wnt1*-Cre (neural crest specific knock out; abbreviated as nc-KO) and *Krt14*-Cre (ectodermal specific knock out; ect-KO) driver lines. (**A**–**D**) Frontal views of conditional allelic series at E17.5. Gray arrows in B–D mark the width noted in A to facilitate visual comparisons. (**E**, **F**) Hematoxylin and eosin stained cryosections at E15.5 show closed and fused palates of conditional mutants at E15.5; no clefting was observed in any conditional mutants (nc-KO: 0/15; ect-KO: 0/8, nc + ect: 0/4). (**G**) Facial width (as measured in Fig. 1E and F) is reduced in conditional nulls; ect-KO: *P* = 0.02, nc + ect-KO: *P* < 0.001) mutants, particularly when both compartments are targeted (nc + ect width: 93% of WT, *P* < 0.001 by one-way ANOVA with Šídák's test). (**H** and **I**) Vomer tail/head ratio (as measured in Fig. 4I and J) is decreased in nc-KO, ect-KO and nc + ect-KO, versus fl/fl control (asterisk denotes *P* < 0.005 by one-way ANOVA with Šídák's MCT versus fl/fl controls). Dashed red line indicates ratio of non-exencephalic *Ranbp1*^−/−^ cohort (from Fig. 4I). Scale bar = 2 mm for A–D.

We next asked whether *Ranbp1* loss of function disrupts a key cellular mechanism that underlies oropharyngeal and craniofacial morphogenesis: interactions between mesenchymal and epithelial cells in the craniofacial primordia. The two tissues that constitute the palatal shelves—mesenchyme primarily derived from cranial neural crest, and an outer epithelial layer derived from the oral ectoderm (25)—both express RANBP1 (see Fig. 3B). Furthermore, the growth, elevation and fusion of the palatal shelves are mediated by reciprocal interactions between epithelium and mesenchyme, via developmental signals that drive cell proliferation to result in directed growth (30). We used a conditional allele for *Ranbp1* to selectively inactivate *Ranbp1* via *Wnt1*-Cre mediated recombination (31) to target neural crest-derived cranial and oropharyngeal mesenchyme (*Ranbp1*^fl/fl^;*Wnt1*-Cre^Cre/+^, abbreviated nc-KO for neural crest conditional Knock Out). To inactivate *Ranbp1* in the ectoderm-derived oral epithelium, we used *Krt14*-Cre (*Ranbp1*^fl/fl^;*Krt14*-Cre^Cre/+^, abbreviated as ect-KO for ectoderm conditional Knock Out) mediated recombination (32). While no Cre-driver is perfectly efficient, *Wnt1*-Cre and *Krt14*-Cre have been used in multiple conditional-knockout models of cleft palate (24,33), and both ablate *Ranbp1* efficiently in their appropriate compartments (Supplementary Material, Fig. S5). In contrast to constitutive *Ranbp1*^−/−^ embryos, nc-KO and ect-KO embryos do not have dramatic gross phenotypes at E17.5 (Fig. 5A–D); none are exencephalic, and overall embryo size appears to be only slightly reduced. Conditional ablation with either or both drivers does not lead to an overt cleft palate or exencephaly (Fig. 5E and F; nc-KO: 0/15; ect-KO: 0/8, nc + ect: 0/4). However, parallel to the midline facial dysmorphology in constitutive *Ranbp1*^−/−^ mutant embryos, facial width is reduced in the tissue-specific loss-of-function mutants, particularly when both compartments are targeted (nc + ect width: 93% of WT, *P* < 0.001 by one-way ANOVA with Šídák's test; Fig. 5G); in contrast, height/width ratios are indistinguishable between all genotypes (*P* > 0.1 for ect-, nc- and nc + ect-KO versus WT as above; not shown). This indicates that *Ranbp1* regulates the capacity of craniofacial mesenchyme and epithelium to interact with one another to elicit appropriate craniofacial morphogenesis.

Similar to their constitutive *Ranbp1*^−/−^ counterparts, conditional *Ranbp1* deletion alters midline cranial bone formation: the vomer is qualitatively and quantitatively dysmorphic in both nc-KO and ect-KO mutants (Fig. 5H and I; nc-KO versus fl/fl: *P* < 0.001, ect-KO versus fl/fl: *P* = 0.003, by one-way ANOVA with Šídák's test). To further evaluate the role of *Ranbp1* in mediating midline craniofacial mesenchymal–epithelial interaction, we asked whether nc + ect-KO, which targets both tissues, enhances the vomer phenotype beyond inactivation in either tissue. This appears to be the case: in double conditional nc + ect-KO vomers, morphology is disrupted versus controls (t/h ratio: 0.78 ± 0.10; *P* = 0.003 versus fl/fl), somewhat more than in single nc-KO and ect-KO conditional *Ranbp1* mutants (Fig. 5H); this difference is distinguishable from the single ect-KO vomer measurements (*P* = 0.005 by *t*-test, but not significant by one-way ANOVA), but not statistically distinguishable from the nc-KO vomer (*P* = 0.2 by *t*-test; see also Supplementary Material, Fig. S6). The morphology of the double-conditional vomer approaches the magnitude observed in constitutive *Ranbp1* nulls without a cleft palate (t/h ratio of 0.78 versus 0.49, respectively), and they are statistically indistinguishable (*P* > 0.5 by Student’s *t*-test; see also Supplementary Material, Fig. S6). Thus, *Ranbp1* contributes to mesenchymal–-epithelial interactions central to the morphogenesis of the midline cranial skeleton.

### *Ranbp1* modulates midline patterning via disruption of BMP signaling

The disruption of the midline cranial skeleton in *Ranbp1*^−/−^ mutants suggests that key midline signaling pathways may be disrupted in the context of *Ranbp1* loss of function. The two most significant signals that pattern the orofacial midline are Sonic Hedgehog (*Shh*) and members of the bone morphogenetic protein family (BMPs). These two signals interact with each other to establish cranial midline patterning (34–36). Mutations in the Shh signaling pathway result in substantial collapse of the craniofacial midline that is more severe than that in *Ranbp1*^−/−^ embryos, including disruption of tissues that are not noticeably altered in *Ranbp1* mutants. Accordingly, we began by assessing genetic interaction between *Ranbp1* and a mutation that enhances midline BMP signaling and diminishes SHH signaling, leading to subtle midline defects (37–39): loss-of-function mutation in the endogenous BMP antagonist Noggin (*Nog*). Like *Ranbp1*^−/−^ mutants, *Nog*^−/−^ mutants have narrow midline facial structures, and like *Ranbp1*^−/−^ mutants, *Nog*^−/−^ embryos die around birth. More significantly, exencephaly and disrupted initial development of the palatal shelves occur in a subset of *Nog*^−/−^ embryos (38) as is the case in *Ranbp1*^−/−^ embryos. Thus, we next asked whether *Nog* and *Ranbp1* interact to enhance phenotypes at the craniofacial midline.

**Figure 6:**
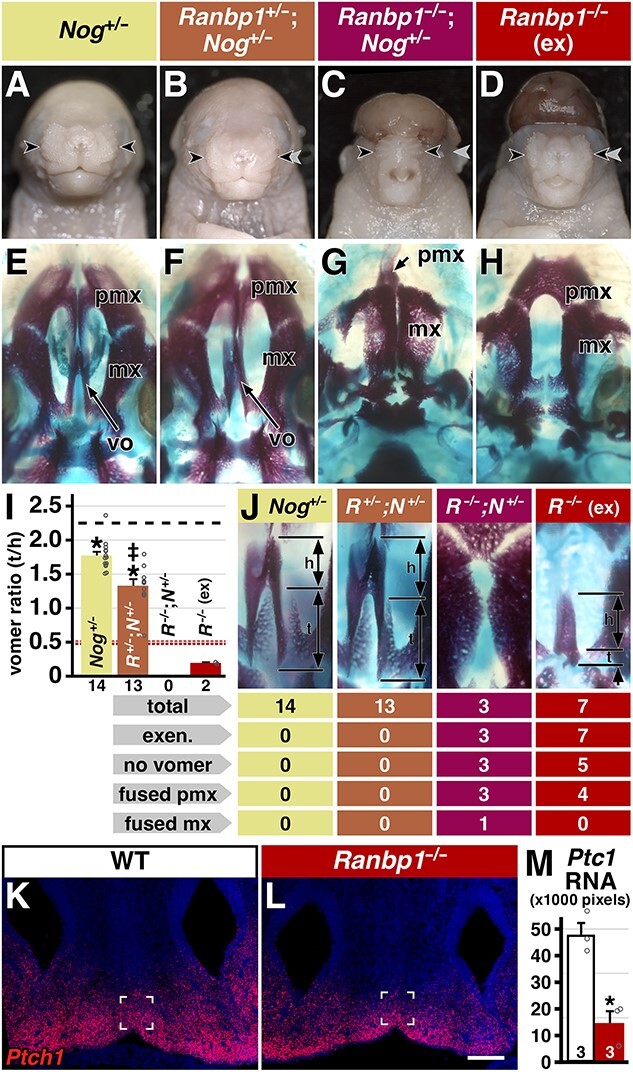
Interaction of *Ranbp1* and *Nog* mutations. (**A**–**D**) Frontal view of *Ranbp1*;*Nog* allelic series at E17.5. Note extreme narrowing of midline facial structures in compound null *Ranbp1*^−/−^;*Nog*^+/−^ (C) and *Ranbp1*^−/−^ (D) (arrows) versus *Nog*^+/−^ (A) and *Ranbp1*^+/−^;*Nog*^+/−^ (B) embryos. Gray arrows in B–D mark the width noted in A to facilitate visual comparisons. (**E**–**H**) Alcian blue/alizarin red staining of upper jaw and palate; note absence of vomer (vo) and collapse of maxilla (mx) and presumptive premaxilla (pmx) in *Ranbp1*^−/−^;*Nog*^+/−^ (K). (**I**) Tail/head ratio is reduced in *Ranbp1*^+/−^, *Ranbp1*^−/−^, and *Ranbp1*^−/−^; *Nog*^+/−^ as compared with WT. Asterisk denotes significance (*P* < 0.001) versus WT by one-way ANOVA with Šídák's MCT across all constitutive alleles used in study; dagger denotes significance (*P* = 0.015) versus *Nog*^+/−^; dashed red line indicates ratio of non-exencephalic *Ranbp1*^−/−^ cohort (from Fig. 4I). (**J**) Representative examples of midline bones for each genotype; frequency of key phenotypes noted below. (**K**–**M**) *Ptc1* expression in E10.5 facial midline by RNAscope *in situ* hybridization, with quantification of labeled pixels shown in (M). Scale bar in P, Q = 0.1 mm; marked box is size of area quantified (see Methods), asterisk denotes significance (*P* = 0.008) by *t*-test.

We generated a series of *Ranbp1*;*Nog* compound mutant embryos to assess the potential interaction between *Ranbp1* and *Nog* function at the developing midline (Fig. 6A–H). *Nog^+/−^* and *Ranbp1*^+/−^;*Nog^+/−^* embryos do not share any gross phenotypes with either *Ranbp1*^−/−^ or *Nog*^−/−^ embryos (Figs 1A, B and2A–C; 38). They are neither exencephalic nor do they have cleft palate. Thus, combined diminished dosage of *Nog* and *Ranbp1* is not sufficient to generate the most severe gross craniofacial dysmorphology associated with full loss of function of either. Nevertheless, *Nog*^+/−^ embryos have a less severe form of the vomer dysmorphology observed in *Ranbp1*^−/−^ embryos (Fig. 6I and J; t/h ratio: 1.77 ± 0.06 versus 1.82 ± 0.04, respectively; *Nog*^+/−^  *P* < 0.0001 versus WT by one-way ANOVA with Šídák’s test). Double-heterozygous *Ranbp1*+/−;*Nog*+/− embryos also have a quantitatively enhanced vomer phenotype versus *Nog*+/− embryos (t/h ratio: 1.35; *P* = 0.0001 versus *Ranbp1*^+/−^, *P* = 0.015 versus *Nog*^+/−^). Thus, quantitative ratiometric analysis of the vomer robustly identifies a targeted disruption of midline craniofacial morphogenesis due to heterozygous *Nog* loss of function as well as interaction between diminished *Ranbp1* and *Nog* dosage.

Finally, we asked whether heterozygous loss of *Nog* modulates *Ranbp1*^−/−^ phenotypes. *Ranbp1*^−/−^;*Nog*^+/−^ compound mutants (Fig. 6C) are profoundly dysmorphic: they are exencephalic, have smaller heads than *Ranbp1*^−/−^ mutants, and in one case a very rare digit deformity (dorsal polydactyly, see Supplementary Material, Fig. S7). *Ranbp1*^−/−^;*Nog*^+/−^ compound mutants also have substantial facial dysmorphology (Fig. 6C), with a compressed face that is narrower than all other genotypes we examined (see Supplementary Material, Figs 8 and 9 for additional detail). This midline compression is even more obvious in the cranial skeleton: in compound mutants the premaxilla is substantially diminished and fused, the vomer is completely absent, and the gap between left and right maxilla is diminished; in one embryo these bones are fully fused (Fig. 6G). Because *Ranbp1*^−/−^;*Nog*^+/−^ compound mutants are all exencephalic, they are most readily compared to the exencephalic subset of *Ranbp1*^−/−^ embryos (Fig. 6D). Exencephalic *Ranbp1*^−/−^ mutants have a more severe form of the partially penetrant midline defects than their non-exencephalic littermates, including a narrow face, premaxilla fusion (4/7 embryos; Fig. 6G), absent vomers (5/7 embryos) and a narrow/arched palate (Supplementary Material, Fig. S9). In the two embryos where a vomer was identified, it was highly dysmorphic (Fig. 6I and J). Thus, each dysmorphic phenotype in the *Ranbp1*^−/−^ cranial skeleton is more severe in *Ranbp1*^−/−^;*Nog*^+/−^ compound mutants. This indicates that *Ranbp1* mutation interacts with *Nog* to modulate craniofacial signaling, particularly along the embryonic craniofacial midline, potentially by disrupting the interactions between *Nog, Shh* and BMPs that establish mediolateral patterning.

To further assess the disruption of midline pattering in *Ranbp1*^−/−^ embryos, we assessed a molecular intermediate of *Shh* signaling that is also a target of BMP signaling levels that are in turn modulated by Noggin: *Ptch1*. *Ptch1* is mediator of *Shh* signaling for craniofacial midline morphogenesis, and is used as an indicator of *Shh* signaling levels (18,40). We quantified expression of *Ptch1* at the midline of the developing oral cavity in E11.5 *Ranbp1*^−/−^ embryos using RNAscope *in situ* hybridization (Fig. 6K–M). *Ptch1* labeling intensity is visibly diminished by *Ranbp1* loss of function (Fig. 6K–L); this was confirmed by quantification of fluorescent puncta, which reflect transcript quantity. Midline transcript levels in *Ranbp1*^−/−^ embryos are 31% of that in the WT (Fig. 6M; *P* = 0.008 by *t*-test, *n* = 3 *Ranbp1*^−/−^, 3 WT). This reduction in *Ptch1* expression parallels to that observed in *Nog*^−/−^ embryos (38), in which Shh signaling is reduced due to the loss of BMP inhibition at the midline. Together these data indicate that *Ranbp1* modulates signaling that mediates midline patterning of craniofacial primordia that underlies appropriate craniofacial differentiation.

### *Ranbp1* ^*+/−*^ phenocopies craniofacial bone anomalies of the *LgDel* 22q11DS model

Null mutants for multiple 22q11.2 candidate genes are associated with craniofacial anomalies (8); however, the extent to which heterozygous deletion of any of these genes contributes to midline cranial skeletal anomalies observed following broader heterozygous 22q11.2 deletion in either humans or mouse models remains unknown. We have shown previously in *LgDel* embryos, in which *Ranbp1* and 27 additional 22q11 orthologs are heterozygously deleted, that palatal shelf elevation is disrupted at low penetrance, and that maxillary differentiation is altered (16). Thus, we asked whether heterozygous *Ranbp1*^+/−^ embryos have similar craniofacial defects to those observed in the *LgDel* mouse.

**Figure 7:**
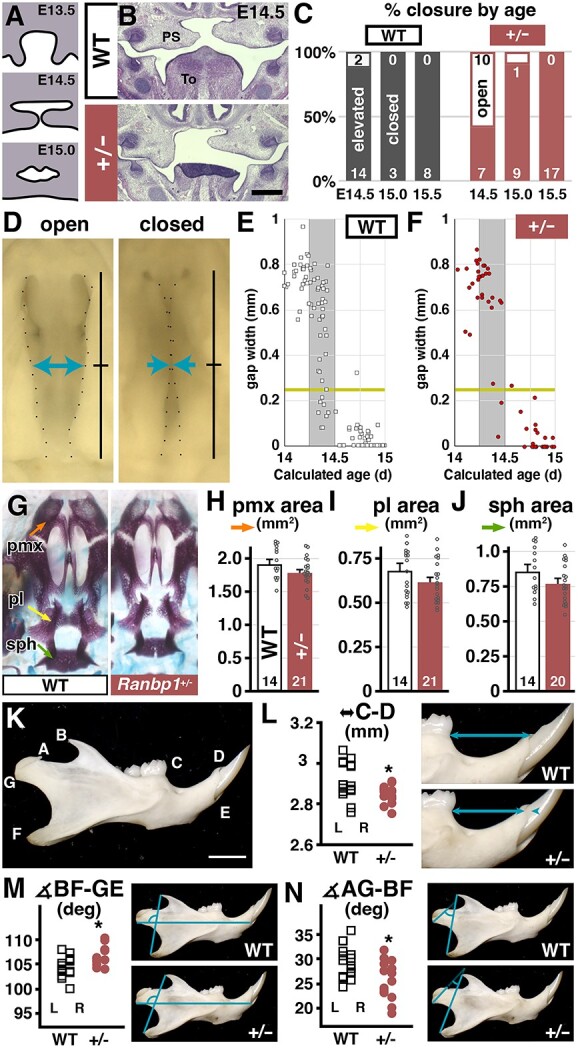
*Ranbp1* heterozygote phenotypes. (**A**–**C**) Palatal shelf elevation/closure is disrupted in *Ranbp1*^+/−^ embryos. (A) Normal timing of palatal shelf closure. (B) Example *Ranbp1*^+/−^ and WT palates at E14.5. (C) Fraction of WT and *Ranbp1*^+/−^ palates matching normal phenotype (elevated at E14.5, closed at E15.0–15.5). *Ranbp1*^+/−^ palate closure is delayed relative to WT (*P* = 0.01 by Fisher’s exact test). (**D**–**F**) Stage corrected quantification of palatal closure. (D) Width of gap between palatal shelves was measured at the midline in fixed and dissected preparations (blue arrows); stage correction was performed by algorithmically assessing photographs of forelimbs (41). (E) Plots of measured gap versus calculated embryonic age. Gray shading notes time when closure typically occurs (E14.25—E14.5); yellow line marks median gap width for WT embryos in cohort. (E, right) Quantification of closure of *Ranbp1*^+/−^ littermates in cohort: average gap in this time window is slightly but not significantly increased (*Ranbp1*^+/−^ = 0.62 mm versus WT = 0.51 mm; *P* = 0.08 by *t-*test); also, more WT palates are closed (separation less than median WT value: WT 9/34, 21%, versus 2/16, 11% for *Ranbp1*^+/−^; *P* > 0.4 by Fisher’s exact test). (**G**–**J**) Assessment of cranial bones in WT and *Ranbp1*^+/−^ head. Measured areas of premaxilla (pmx, H), palatine (pl, I) and basisphenoid (bs, J) are slightly smaller. All measures assessed together are significant (by two-way ANOVA; measure × genotype; genotype *P* = 0.007); however, individual measures (when corrected for FDR) are not. (**K**–**N**) Jaw morphology is altered in *Ranbp1*^+/−^ adults at P40. Measurements were made between cardinal points (K) on both left and right mandibles; significant differences were observed for anterior jaw length (L, *P* = 0.016, *n* = 8 WT/8 *Ranbp1*^+/−^), angle of posterior jaw (M, *P* = 0.005, *n* = 8/7) and angle of condyloid process (N, *P* = 0.010, *n* = 8/7), as assessed by two-way ANOVA (jaw side × genotype).

WT palatal shelf elevation and fusion follows a stereotyped time scale (Fig. 7A): by E13.5, the palatal shelves grow out from the dorsolateral oral cavity, elevate and grow together so that by E14.5, they are typically elevated and nearing fusion and closure. By E15, the palatal shelves are typically closed, and by E15.5, little sign of the fusion process remains. We compared this schedule of palatal elevation and fusion in sections through the pharyngeal region in *Ranbp1*^+/−^ versus WT embryos (Fig. 7B). We scored whether *Ranbp1*^+/−^ palatal shelves were properly elevated (E14.5) or closed (E15.0–15.5) as they are in the WT at these ages (Fig. 7C). Palatal shelves in *Ranbp1*^+/−^ embryos tend to elevate later than their WT littermates (*Ranbp1*^+/−^ = 7/17, or 41% elevated by E14.5; versus 12/14, or 85% elevated for WT, *P* = 0.01 by Fisher’s exact test), suggesting a variably penetrant delay in palate morphogenesis due to heterozygous *Ranbp1* mutation. By E15, however, most *Ranbp1*^+/−^ palates are closed (8/9), and all appear superficially normal by E15.5 (17/17). This change was slight; thus, we reevaluated palatal closure using limb morphology as an independent metric of embryonic developmental stage (41), by measuring the distance between palatal shelves at the midpoint in embryos staged based upon limb morphogenesis (Fig. 7D). Using this approach to compare similar developmental stages, the delay in palatal closure is less robust (Fig. 7E and F). There is a general trend for delay in the *Ranbp1*^+/−^ embryos, but it is no longer statistically significant (*Ranbp1*^+/−^: 2/16 closed = 12.5%; versus WT: 9/34 closed = 26.4% for embryos identified as gestational E14.25–E14.5 based upon forelimb differentiation; Fig. 7E and F). At later stages, *Ranbp1* heterozygous deletion also appears to impact the development of the cranial skeleton. Although the bone structure at E17.5 is relatively normal (Fig. 7G), there appears to be a modest reduction in cranial bone area (Fig. 7H–J) that parallels the more dramatic reductions seen in their null mutant littermates (Fig. 2E–G). Trends were seen for smaller areas in individual measurements of the premaxilla (Fig. 7H), palatine (Fig. 7I) and sphenoid (Fig. 7J), which are significant when combined (*P* = 0.007 by two-way ANOVA). Thus, heterozygous mutation of *Ranbp1* does not significantly disrupt palatal closure; nevertheless, *Ranbp1* may have a modest, variable impact on the timing of palatal elevation and cranial bone morphology.

We next asked whether there might be similar changes in facial bone structure in adult *Ranbp1*^−/−^ mice. We previously reported jaw dysmorphology in adult *LgDel* mice (16,17), consistent with mild micrognathia that is commonly observed in 22q11DS (4). Furthermore, we found occasional instances of more severe midline craniofacial truncation and asymmetry using *in vivo* fluorographic imaging in adult *LgDel* mice (17). To determine whether similar changes occur in adult *Ranbp1*^+/−^ mice, we measured distances and angles between cardinal points on the jaws of young adult WT and *Ranbp1*^+/−^ mice (Fig. 7K). We found statistically significant declines in the length of the anterior jaw (Fig. 7L, ‘C-D’ measure, *P* = 0.016), the angle of the posterior jaw (the position of the coronoid process/angle relative to the A–P axis of the jaw, Fig. 7M, *P* = 0.005) and the angle of the head of the jaw (condyloid process relative to the coronoid process/angle, Fig. 7N, *P* = 0.010). Together, these dysmorphologies suggest that the *Ranbp1*^+/−^ jaw has multiple features that define mild micrognathia, similar to that in the *LgDel* jaw, as well as a slight upward curve of the lower jaw relative to WT littermates. We did not find examples in the *Ranbp1*^+/−^ cohort of the more severe (but rare) craniofacial defects or asymmetry occasionally observed in the *LgDel* mouse (17). Finally, we also asked if *Ranbp1* heterozygotes have defects in growth/weight gain as observed in *LgDel* pups (16); however, such a growth deficit was not observed (Supplementary Material, Fig. S10). Thus, *Ranbp1*^+/−^ mice apparently have some, but not all, of the mild craniofacial anomalies that parallel those associated with broader 22q11 gene deletion in *LgDel* mice.

**Figure 8:**
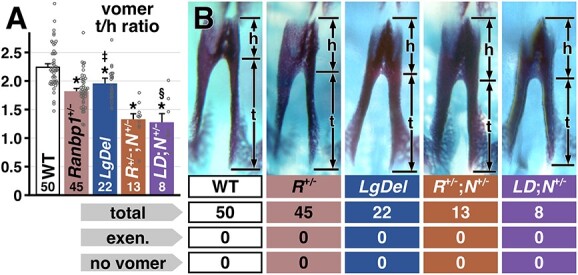
Vomer phenotype in *Ranbp1*^+/−^ is indistinguishable from phenotype of *LgDel*. (**A**) Vomer tail/head ratio was calculated for WT, *Ranbp1*^+/−^, *LgDel*, as well as *Ranbp1*^+/−^;*Nog* and *LgDel*;*Nog* compound heterozygotes. *Ranbp1*^+/−^ and *LgDel* vomers are mildly dysmorphic (* indicates *P* < 0.005 versus WT, by one-way ANOVA with Šídák's MCT), and are indistinguishable from each other (‡ indicates *P* > 0.96 versus *Ranbp1*^+/−^). *Ranbp1*^+/−^ and *LgDel* phenotypes are both enhanced by *Nog*^+/−^; compound heterozygotes are significantly different from WT (* indicates *P* < 0.005), and significantly different from *Ranbp1*^+/−^ single heterozygotes (*P* < 0.005); compound heterozygotes are also statistically indistinguishable from each other (§ indicates *P* > 0.99 versus *Ranbp1*^+/−^;*Nog*^+/^). (**B**) Examples of genotypes quantified in (A); horizontal marks are used to illustrate how proportions of mutant vomers compared with WT example.

Finally, to determine if heterozygous *Ranbp1* mutation phenocopies specific *LgDel* midline cranial skeletal dysmorphology, we assayed midline craniofacial bone development using our ratiometric analysis of vomer morphology in both *Ranbp1*^+/−^ and *LgDel* E17.5 embryos. *Ranbp1*^+/−^ vomers have a milder form of the dysmorphology observed in *Ranbp1*^−/−^ (t/h ratio for WT: 2.25, het: 1.82; *P* < 0.0001; *n* = 50 WT, 45 het; Fig. 8A and B). *LgDel* vomers are also dysmorphic (t/h ratio *LgDel*: 1.92; n = 22; *P* = 0.003 versus WT; Fig. 8A), and the magnitude of dysmorphology is virtually identical to the single-gene *Ranpb1*^+/−^ heterozygote: the *Ranbp1*^+/−^ t/h ratio is 81.1% of WT; *LgDel* is 85.7%, and the two populations are statistically distinct from WT, but not from each other (*Ranbp1*^+/−^ versus *LgDel P* = 0.18 by *t*-test; see also Supplementary Material, Fig. S11). Apparently, heterozygous loss of *Ranbp1* function is the monogenic basis of this robustly quantifiable phenotype. To further test this conclusion, we asked whether *LgDel* vomer differentiation is also modulated by heterozygous loss of *Nog,* as is seen with *Ranbp1*^+/−^;*Nog*^+/−^ embryos (see Fig. 6). This is indeed the case: *LgDel*;*Nog*^+/−^ compound embryos have a vomer phenotype that is statistically indistinguishable from the *Ranbp1*^+/−^;*Nog*^+/−^ embryos (t/h ratio for *Ranbp1*^+/−^;*Nog*^+/−^: 1.34 ± 0.08 versus *LgDel*;*Nog*^+/−^: 1.28 ± 0.14; both significant by one-way ANOVA with Šídák's test versus WT at *P* < 0.0001; whereas *Ranbp1*^+/−^;*Nog*^+/−^ versus *LgDel*;*Nog*^+/−^ are indistinguishable at *P* = 0.68 by *t*-test). Thus, for this quantitative midline craniofacial phenotype, *Ranbp1* mutation, via its interaction with the network of signaling pathways at the cranial midline that includes BMP-*Shh*-*Nog*, appears to be a monogenic driver of a subset of the cranial skeletal dysmorphology associated with 22q11.2 deletion.

## Discussion

We have defined *Ranbp1* as a major contributor to dosage dependent disruption of palatal, oropharyngeal skeletal and midline craniofacial morphogenesis in the context of broader 22q11 gene deletion. It has long been a goal to ascribe individual 22q11DS phenotypes to specific candidate genes; however, except for the identification of *Tbx1* as a primary heterozygous contributor to aortic arch dysmorphology in 22q11DS (14,15), this goal has been elusive. Homozygous loss-of-function mutations of several 22q11.2 candidate genes are associated with severe dysmorphology in humans or mouse models; however, relatively few have been shown singularly responsible for heterozygous phenotypes (8). This is primarily due to a fundamental challenge to identifying the nature of heterozygous phenotypes (both in humans and mice)—heterozygous phenotypes are generally subtle and highly variable in comparison to phenotypes observed in null mutants, as is clearly the case for *Ranbp1* mutations. Our study, therefore, began by identifying multiple clear phenotypes in homozygotes, and then establishing robust quantification methods for assessing whether such phenotypes were reflected in the highly variable *Ranbp1*^+/−^ or *LgDel* populations. We found that homozygous loss-of-function mutations in *Ranbp1* result in robust, quantifiable, highly penetrant craniofacial phenotypes that parallel those observed in broader 22q11 deletion, including multiple assessments of facial narrowing, palatal clefting and facial bone dysmorphology. Of these phenotypes, a subset could be quantifiably identified in *Ranbp1* heterozygotes and in the *LgDel* mouse model of 22q11DS. Thus, our results provide statistically robust, anatomically specific evidence for the contribution of a single 22q11 deleted gene, *Ranbp1*, to midline craniofacial dysmorphology seen due to broader 22q11 deletion.

**Figure 9:**
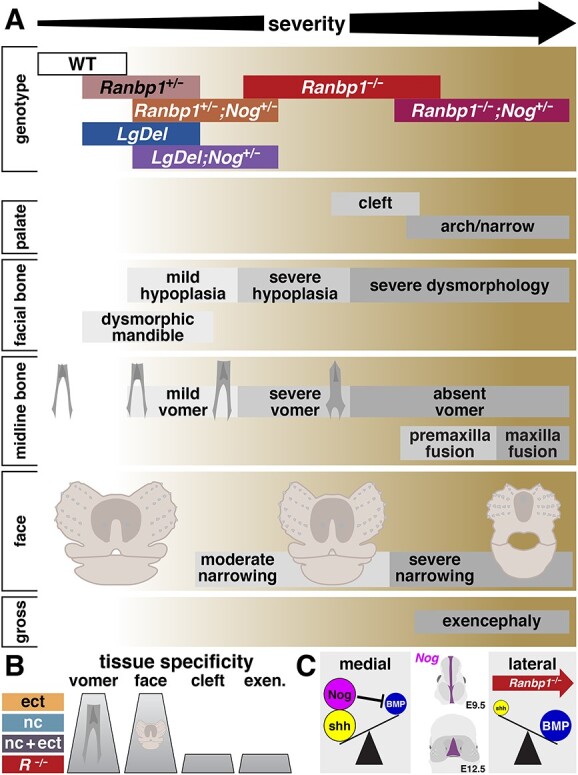
Summary of key findings. (**A**) Alignment of key phenotypes with genotypes in which they occur. As phenotypes are variable within each genotype, the width of the bar in the genotype row corresponds to the range of severity that was observed, as illustrated for each of the key phenotypes. (**B**) Conditional *Ranbp1*^−/−^ mutants show subsets of key phenotypes, indicating tissue specificity. As noted, conditional ablation of *Ranbp1* in ectoderm (ect, Krt14-cre;*Ranbp1*^fl/fl^), neural-crest derived facial mesenchyme (nc, Wnt1-cre;*Ranbp1*^fl/fl^), or in both ectodermal and crest compartments (nc + ect) lead to cranial bone (vomer) and facial width (face) phenotypes; however, more severe phenotypes such as cleft palate and exencephaly only appear in constitutive *Ranbp1*^−/−^ mutant embryos. (**C**) Schematic of hypothesized signaling changes in *Ranbp1*^−/−^ embryos. In the developing face, BMPs are lateralizing signals, while *Shh* is medializing. *Nog* is expressed along the midline of the developing head at E9.5, and along the midline of the developing frontonasal region; this midline-expressed *Nog* further enhances medialization by inhibiting BMP. Loss of *Ranbp1* acts to disrupt this midline patterning, thus acting as a lateralizing signal, in a manner complementary to the lateralization that occurs in *Nog* mutant embryos. *Nog* expression schematic adapted from ref. (38); *Shh*/BMP ‘see-saw’ motif adapted from ref. (57).

### *Ranbp1* and *LgDel* mice mirror a subset of 22q11DS palate and orofacial defects

Palatal defects have long been considered a central focus of the spectrum of anomalies associated with 22q11DS. Overt cleft palate is present in a significant number of individuals with 22q11DS (~11%; 4); therefore, it is of significant interest that *Ranbp1* null mutants have a partially penetrant cleft palate phenotype. This is not unique among 22q11DS candidate genes; *Tbx1* null mice also have cleft palates (13). Nevertheless, heterozygous deletion of either *Tbx1* or *Ranbp1* does not lead to an overt cleft (nor does compound *Tbx1*/*Ranbp1* heterozygosity). Likewise, clefting is not observed in the *LgDel* model of 22q11DS, which models numerous other key phenotypes (8,42). Thus, it is likely that mouse models do not accurately model this modestly penetrant human phenotype. There are significant structural differences between the mouse and human palate; for example, the murine premaxilla and maxilla bones do not form a fully closed hard palate. Some midline oropharyngeal structures such as the uvula and its associated muscles (musculus uvulae) are not present in mouse, and the mouse head is proportionally elongated relative to the human. Thus, it is not necessarily surprising that there are differences between humans and mouse models in some phenotypic domains.

While overt clefting is obviously of great interest, even more individuals with 22q11DS (>70%) have milder but clinically significant anomalies in palate structure and/or function (43) such as a submucosal cleft palate or anomalies in midline structures or disrupted movement of palatal muscles. We found evidence of disruptions to the palate and other key orofacial structures based upon morphometrically quantified dysmorphology of the facial bones from large samples of multiple related genotypes (Fig. 9A). The bones that contribute to the hard palate and upper jaw—premaxilla, maxilla and palatine—along with the sphenoid bones are clearly hypomorphic and/or dysmorphic in *Ranbp1*^−/−^ embryos, and a subset are modestly but statistically hypomorphic in heterozygotes as well. Additionally, adult *Ranbp1*^+/−^ mice have statistically significant (although visually subtle) lower jaw dysmorphologies that mirror the micrognathia associated with 22q11DS (4). Although these facial dysmorphologies are relatively subtle and variable compared with their null mutant littermates, it should be noted that these heterozygous phenotypes parallel craniofacial and oropharyngeal anomalies observed in the human 22q11DS cohort (4).

The most robust evidence for facial dysmorphology we identified in multiple mouse models of 22q11 deleted and related genes is not in the palate, but rather in the only unpaired bone found in the prenatal mouse (and human) midline facial skeleton: the vomer. The quantitatively robust dysmorphology of the vomer in *Ranbp^−/−^, Ranbp1^+/−^* and *LgDel* embryos provides a reliable focal assay of broader palatal and midline orofacial dysmorphology that results from these genomic lesions. Vomer deformity has been considered a hallmark of submucosal cleft palate in radiological imaging studies (44–46), and deformities are identifiable in both overt and submucosal cleft palate—the most common 22q11DS oropharyngeal/craniofacial deficit. Thus, it is likely that these facial bone anomalies are hallmarks of oropharyngeal dysmorphology that may contribute to impairments such as velopharyngeal insufficiency observed frequently in individuals with 22q11DS (4). Our analysis provides a foundation for new imaging studies to better characterize the underlying cranial skeletal correlates of oropharyngeal dysmorphology and dysfunction at multiple stages in individuals with 22q11DS.

### Midline dysmorphology is a central 22q11DS craniofacial phenotype

The human spectrum of common 22q11DS craniofacial phenotypes includes facial features consistent with midline defects, including hypertelorism, malar flatness and nasal dimpling (4); however, there has been little direct evidence of specific midline patterning defects. Examination of the *Ranbp1* allelic series provides robust evidence of deficient midline development: In the context of this full allelic series, it becomes clear that there is a spectrum of midline phenotypes, beginning with mild vomer dysmorphology and facial bone hypoplasia that is visible in *Ranbp1*^+/−^ and *LgDel* embryos, progressing into more severe dysmorphology including cleft palate, absence/fusion of midline bones and visible facial narrowing as observed in *Ranbp1*^−/−^ mutants (Fig. 9A). Compound mutations of *Ranbp1*^−/−^ and the heterozygous mutation of a key midline signal, Noggin, make this midline collapse even more obvious, leading to complete loss of midline structures (loss of vomer, premaxilla and maxilla fusion) and severe narrowing of facial structures.

Our evidence suggests a requirement for *Ranbp1* to execute successfully additional cellular and molecular processes that underlie craniofacial development. These include the mesenchymal/epithelial interactions that drive craniofacial differentiation and coordinate midline patterning beginning at the earliest stages of head closure, particularly those mediated by BMP as well as SHH signaling, which are complemented by additional signals including FGF8 and retinoic acid (47–50). Nevertheless, the role of *Ranbp1* is not likely limited to mesenchymal–epithelial interactions. The absolute range of dysmorphologies seen in constitutive nulls (including cleft palate and exencephaly) are not completely recapitulated in the tissue specific conditional mutants (Fig. 9B). This may reflect inefficiency of the Cre drivers we used; however, it is possible that additional influences of *Ranbp1* in other tissues contribute to craniofacial differentiation. For example, Shh expressed by the prechordal plate during early embryogenesis (51) acts to pattern the ventral forebrain epithelium, which in turn acts as a midline source of Shh during later stages of facial development (52); our conditional mutants would presumably not target any of these tissues. Facial midline development is primarily established by the balance of *Shh* originating from midline structures, versus BMP signaling that acts to lateralize facial structures (Fig. 9C). Additional signals act to modulate this balance, including the midline expression of Noggin which further suppresses BMP signaling. Apparently, *Ranbp1* is an enhancer of midline signaling; its absence lateralizes facial structures. Ranbp1 mediates nucleocytoplasmic trafficking, which is central to the function of most developmental signaling pathways including BMPs, *Shh*, FGFs and other developmental signals utilized in facial morphogenesis. The phenotypes we identify in a range of *Ranbp1* single, conditional and compound mutants are consistent with this primary function of the RANBP1 protein.

### Contiguous gene effects versus gene network drivers in copy number variant disorders

We have shown that *Ranbp1* influences cellular and developmental mechanisms that underlie midline craniofacial differentiation, in addition to its influences on hindbrain patterning, cranial nerve differentiation and cerebral cortical neurogenesis (19,20). In each instance, *Ranbp1* homozygous loss of function leads to severe, highly penetrant phenotypes that target the same structures, cells and molecular mechanisms compromised by broader 22q11 deletion in *LgDel* mice. Nevertheless, our quantitative analyses of phenotypic severity and penetrance in *Ranbp1* heterozygotes do not demonstrate the sort of linear equivalence that defines a classical ‘contiguous gene syndrome’ (53), where a set of unrelated candidate genes independently contribute to phenotypes associated with the syndrome. While some *Ranbp1*^+/−^ and *LgDel* phenotypes are closely matched (vomer and mandible morphology), heterozygous loss of *Ranbp1* clearly does not recapitulate the whole spectrum of 22q11DS craniofacial/oropharyngeal-related phenotypes. For example, *Ranbp1* heterozygotes do not display multiple phenotypes observed in *Lgdel* mice, including functional deficiencies in suckling, feeding and swallowing (16,17), including the significant delay in postnatal weight gain seen in *LgDel* pups that mirrors that observed in human infants with 22q11DS (16, Supplementary Material, Fig. S6).

It is not yet clear whether the suckling, feeding and swallowing impairments that accompany 22q11 deletion are primarily due to structural dysmorphology, or are instead a consequence of aberrant neural development, such as the disrupted cranial sensory innervation (16,54) and aberrant brainstem motor neuron function (55) observed in the *LgDel* mouse. *Ranbp1*^−/−^ mutants do have hindbrain patterning disruptions and cranial nerve dysmorphology that are similar to, but more severe than those seen in *LgDel* (16,19); however, neither consistent hindbrain patterning changes nor statistically significant cranial nerve disruptions are seen in *Ranbp1*^+/−^ embryos. Thus, *Ranbp1* is unlikely to be the singular contributor to oropharyngeal dysfunction, including disrupted suckling, feeding and swallowing, due to broader 22q11 gene deletion.

Our evidence for *Ranbp1*, as well as that from other studies of other single 22q11 genes (8), suggests that for 22q11 deletion, independent and separable ‘contiguous’ or single gene phenotypes are rare. Instead, obligate functions of multiple 22q11 genes, assessed by full loss-of-function mutations, implicate many of these genes in developmental mechanisms relevant to 22q11DS phenotypes, even though heterozygous deletion alone of the same genes does not yield identical changes in developing or mature 22q11-deleted mice. Instead, we propose that *Ranbp1* and other single 22q11 genes are ‘drivers’ of phenotypic change that engage broader, overlapping gene networks that are sensitive to dosage changes of additional 22q11 genes. Thus, even when genetic background is held constant, as in our mouse experiments, penetrance, severity and phenotypic variability remain quantitatively distinct in the full 22q11 deletion model compared to single 22q11 gene heterozygous mutants. This ‘gene-network-driver’ interpretation still places primary responsibility for 22q11DS phenotypes with the causal deletion. Nevertheless, it provides a framework for considering how context-dependent interactions or individual genetic background, including otherwise benign polymorphisms, might further influence gene networks anchored by multiple 22q11 gene ‘drivers’ to yield variable outcomes seen in every 22q11DS phenotypic domain.

## Materials and Methods

### Animals

All mouse lines are carried on a C57/BL6N background (Charles River Labs, Wilmington, MA). The constitutive alleles for *Ranbp1* (20), *LgDel* (15), *Tbx1* (13) and *Nog* (56) have all been maintained on a C57/BL6N background for at least 10 generations (>30 for *LgDel* and *Tbx1*). The *Ranbp1* conditional allele [C57BL/6 N-*Ranbp1* < tm1a(KOMP)Wtsi>/Tcp] was acquired from The Centre for Phenogenomics, Toronto, Canada; the targeting cassette was excised by crossing to an FLP deleter strain produce an allele with exon 3 of *Ranbp1* flanked by LoxP sites. Cre-expressing strains were obtained from Jackson Laboratories (Bar Harbor, ME; *Wnt1*-Cre, #009107; *Krt14*-Cre, #018964). The George Washington University Institutional Animal Care and Use Committee (IACUC) reviewed and approved all animal procedures.

### Histology and scanning electron microscopy

For section imaging, embryos were fixed in 4% paraformaldehyde, and cryosectioned at 10 μm for hematoxylin and eosin (H&E) staining, or at 12 μm for immunofluorescent analysis. Primary antibodies used were rabbit anti-Phospho-Ser10-Histone H3 (PH3; Cell Signaling Technologies, Danvers, MA, #9701, 1:100 dilution), mouse anti- 5-bromo-2′-deoxyuridine (BrdU; BD Biosciences, Franklin Lakes, NJ, #555627, 1:100) or rabbit anti-RANBP1 (Abcam, Cambridge, UK, #ab97659, 1:4000). RNA in situ hybridization analysis was performed using RNAscope to visualize *Ranbp1* message (Advanced Cell Diagnostics, Newark, CA, #NPR-0009145). For scanning electron microscopy, E17.5 embryos were fixed in glutaraldehyde and dissected to remove lower jaw from head to expose the palate. Embryos were then prepared for imaging with assistance from the GWU Nanofabrication and Imaging Core by treating with uranium acetate, dehydrating and sputter coating, and then imaged on an FEI Teneo LV SEM (Thermo Fisher Scientific, Waltham, MA).

Embryonic bone preparations were made from well-fixed embryos (>24 h in paraformaldehyde) by dehydrating embryos in alcohol, extracting lipid for >48 h in acetone, staining with 0.05% Alizarin red/0.025% Alcian blue/0.87 M acetic acid in 70% EtOH for > 48 h at 40°C, then destaining and clearing with 2% potassium hydroxide followed by equilibrating in 75% glycerol. Adult bone preparations were prepared by digestion for several days in a proteinase K solution as described previously (17).

### Measuring and statistics

Measurements of bone length were performed as described above, on digital images of bones taken with a Leica (Wetzlar, Germany) M480 with a 5 MP camera, and normalized to a scale bar imaged in the same session. To prevent repeated analysis of sample sets that are compared across multiple figures, we combined all results with constitutive alleles into a meta dataset, which was analyzed by one-way ANOVA with Holm–Šídák's MCT via Prism software (Graphpad, San Diego, CA) to characterize differences between individual genotypes. For the stage-corrected measure of palatal closure, embryos were harvested at E14.5 ± 0.25 days, and the developmental age was calculated using an independent measure of embryonic age (EMOSS) based on limb morphology (41). The lower jaw was removed, and the palatal shelves imaged in whole-mount preparations. The distance between the palatal shelves was measured at the midpoint of the palate (D, blue arrows); this was correlated with the calculated embryonic age. For assessment of ‘open’ versus ‘closed’ in this assay, the median value of the WT cohort (0.25 mm) was used. For quantifying RNAscope images, samples were processed and imaged as matched pairs with identical hybridization and imaging conditions. Confocal images at 40× magnification were then quantified by selecting a 600 × 600-pixel ROI at the midline, using ImageJ to set a threshold where labeled puncta were visible for both samples, and quantifying the pixels occupied by labeled puncta; significance between genotypes was calculated by paired *t*-test via Excel (Microsoft, Redmond, WA). All other tests were performed as described in the text, using GraphPad Prism for calculations.

## Supplementary Material

Ranbp1_Supplemental_Figures_1_ddad030

Ranbp1_Supplemental_Figures_2_ddad030

Ranbp1_Supplemental_Figures_3_ddad030

Ranbp1_Supplemental_Figures_4_ddad030

Ranbp1_Supplemental_Figures_5_ddad030

Ranbp1_Supplemental_Figures_6_ddad030

Ranbp1_Supplemental_Figures_7_ddad030

Ranbp1_Supplemental_Figures_8_ddad030

Ranbp1_Supplemental_Figures_9_ddad030

Ranbp1_Supplemental_Figures_10_ddad030

Ranbp1_Supplemental_Figures_11_ddad030

## Data Availability

The data generated for this paper will be available from the corresponding author on reasonable request.
