# Supplementary material for: Ranbp1 modulates morphogenesis of the craniofacial midline in mouse models of 22q11.2 deletion syndrome
Source: Hum Mol Genet. 2023 Feb 15;32(12):1959–74. doi: 10.1093/hmg/ddad030 (PMC10244217; doi:10.1093/hmg/ddad030)
Supplement: Ranbp1_Supplemental_Figures_1_ddad030 [file ranbp1_supplemental_figures_1_ddad030.pdf]

Supplemental Figure 1

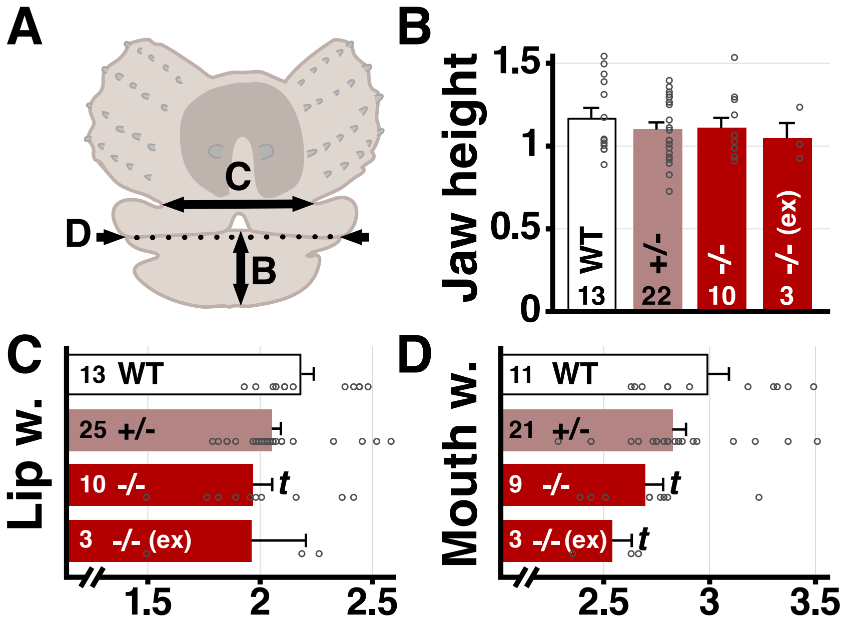

Supplemental Figure 1. Additional measures of facial morphology in *Ranbp1* mutant embryos at E17.5. (A) Illustration of measurement landmarks for lower jaw height (B), width of upper lip/bottom of whiskers (C), and width across mouth opening (D). These measures are more variable than the two featured in Fig. 1, but an analysis uncorrected for multiple comparisons (T-test) follow the same general trend: The vertical measure (Jaw height, B) is not significantly different for non-exencephalic nulls (vs. WT; P=0.51 by Student's T-test, respectively). In contrast, horizontal measures for null embryos trend towards smaller features, for both lip width (C; P=0.04) and mouth width (D; P=0.03). Fewer exencephalic samples were measured, but a parallel trend is also apparent (vertical: jaw height P=0.41; horizontal: lip width P=0.17, and mouth width P=0.04).
