# Supplementary material for: Ranbp1 modulates morphogenesis of the craniofacial midline in mouse models of 22q11.2 deletion syndrome
Source: Hum Mol Genet. 2023 Feb 15;32(12):1959–74. doi: 10.1093/hmg/ddad030 (PMC10244217; doi:10.1093/hmg/ddad030)
Supplement: Ranbp1_Supplemental_Figures_2_ddad030 [file ranbp1_supplemental_figures_2_ddad030.pdf]

Supplemental Figure 2

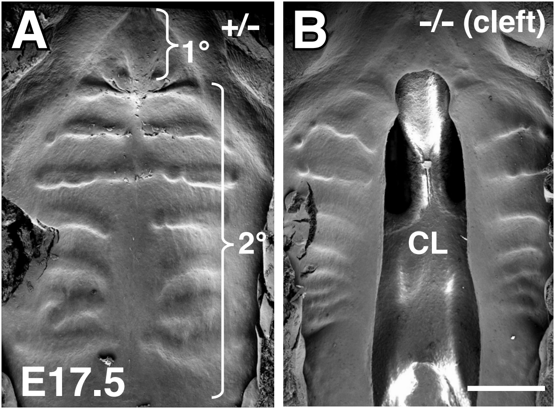

Supplemental Figure 2. SEM of sample E17.5 palates. (A) Normal morphology in a heterozygote embryo. (B) Overt cleft palate without cleft lip in an null mutant embryo. Note intact primary (1°) and secondary (2°) palate in (A); while in (B), *Ranbp1*$^{-/-}$ palate displays overt cleft (CL) across entire secondary palate while sparing primary palate. Scale bar = 1 mm.
