# Supplementary material for: Ranbp1 modulates morphogenesis of the craniofacial midline in mouse models of 22q11.2 deletion syndrome
Source: Hum Mol Genet. 2023 Feb 15;32(12):1959–74. doi: 10.1093/hmg/ddad030 (PMC10244217; doi:10.1093/hmg/ddad030)
Supplement: Ranbp1_Supplemental_Figures_3_ddad030 [file ranbp1_supplemental_figures_3_ddad030.pdf]

Supplemental Figure 3

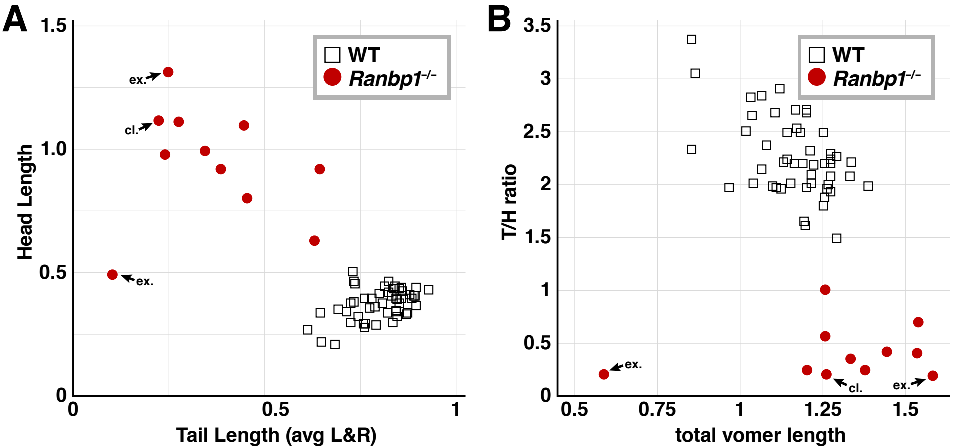

Supplemental Figure 3. Alternate visualizations of vomer morphology demonstrate clear distinction between WT and *Ranbp1*$^{-/-}$ specimens. (A) Plot of WT and *Ranbp1*$^{-/-}$ vomer head vs. tail lengths illustrates that the vomers of null embryos are morphologically distinct from those of WT littermates. (B) Plot of vomer Tail/Head (T/H) ratio vs. total vomer length illustrates that the T/H ratio of null embryos is distinct from WT littermates, regardless of total vomer size.
