# Supplementary material for: Ranbp1 modulates morphogenesis of the craniofacial midline in mouse models of 22q11.2 deletion syndrome
Source: Hum Mol Genet. 2023 Feb 15;32(12):1959–74. doi: 10.1093/hmg/ddad030 (PMC10244217; doi:10.1093/hmg/ddad030)
Supplement: Ranbp1_Supplemental_Figures_4_ddad030 [file ranbp1_supplemental_figures_4_ddad030.pdf]

Supplemental Figure 4

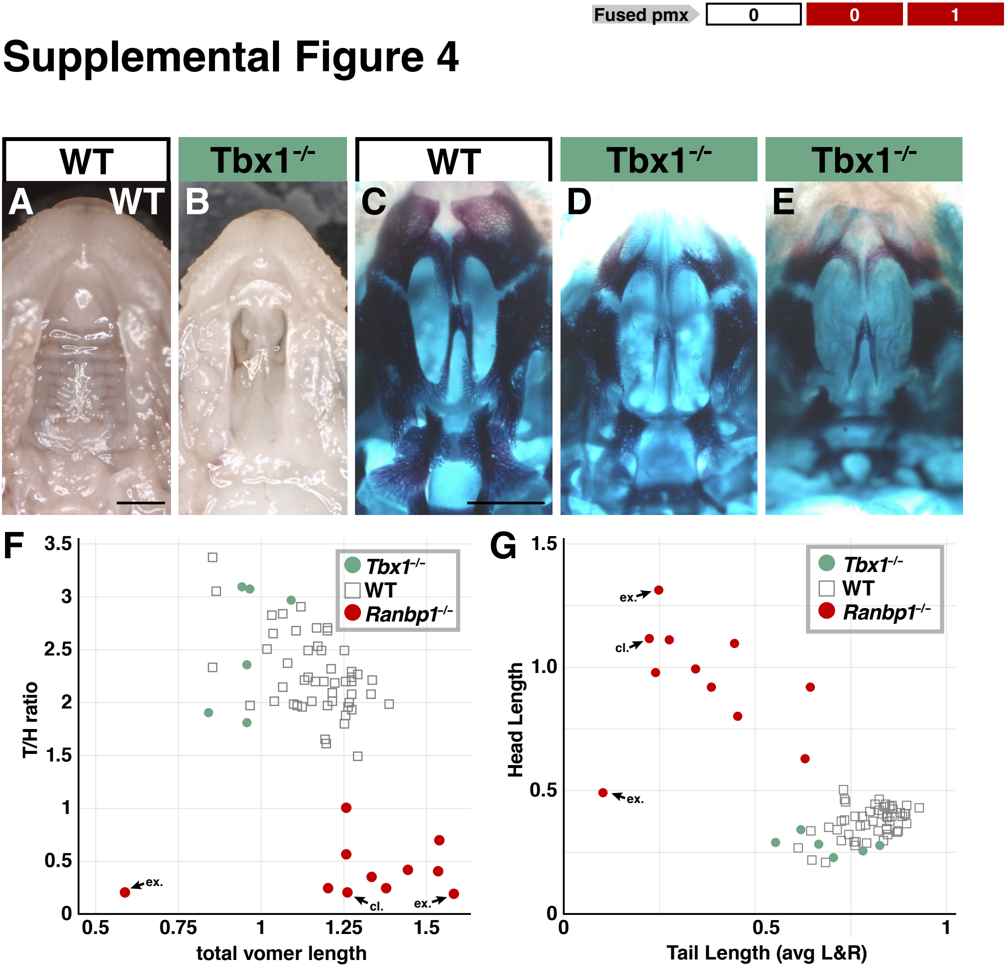

Supplemental Figure 4. (A-B) Palates of WT and *Tbx1*$^{-/-}$ embryos at E17.5, illustrating overt cleft palate observed in *Tbx1*$^{-/-}$ mutants. (C-E) Bone morphology of E17.5 WT and two sample *Tbx1*$^{-/-}$ embryos. There is general hypertrophy of facial bones in the *Tbx1*$^{-/-}$ embryos; however, thte vomer is present in all *Tbx1*$^{-/-}$ embryos and has similar proportions to that of WT embryos. (F-G) Alternate visualizations of vomer morphology demonstrate similarity between WT and *Tbx1*$^{-/-}$ specimens, as in Supp. Fig. 3; both plots illustrate that the vomers of *Tbx1*$^{-/-}$ embryos are morphologically similar to those of WT embryos and distinct from *Ranbp1*$^{-/-}$ embryos, regardless of total vomer size. Scale bars = 1mm.
