# Supplementary material for: Ranbp1 modulates morphogenesis of the craniofacial midline in mouse models of 22q11.2 deletion syndrome
Source: Hum Mol Genet. 2023 Feb 15;32(12):1959–74. doi: 10.1093/hmg/ddad030 (PMC10244217; doi:10.1093/hmg/ddad030)
Supplement: Ranbp1_Supplemental_Figures_5_ddad030 [file ranbp1_supplemental_figures_5_ddad030.pdf]

Supplemental Figure 5

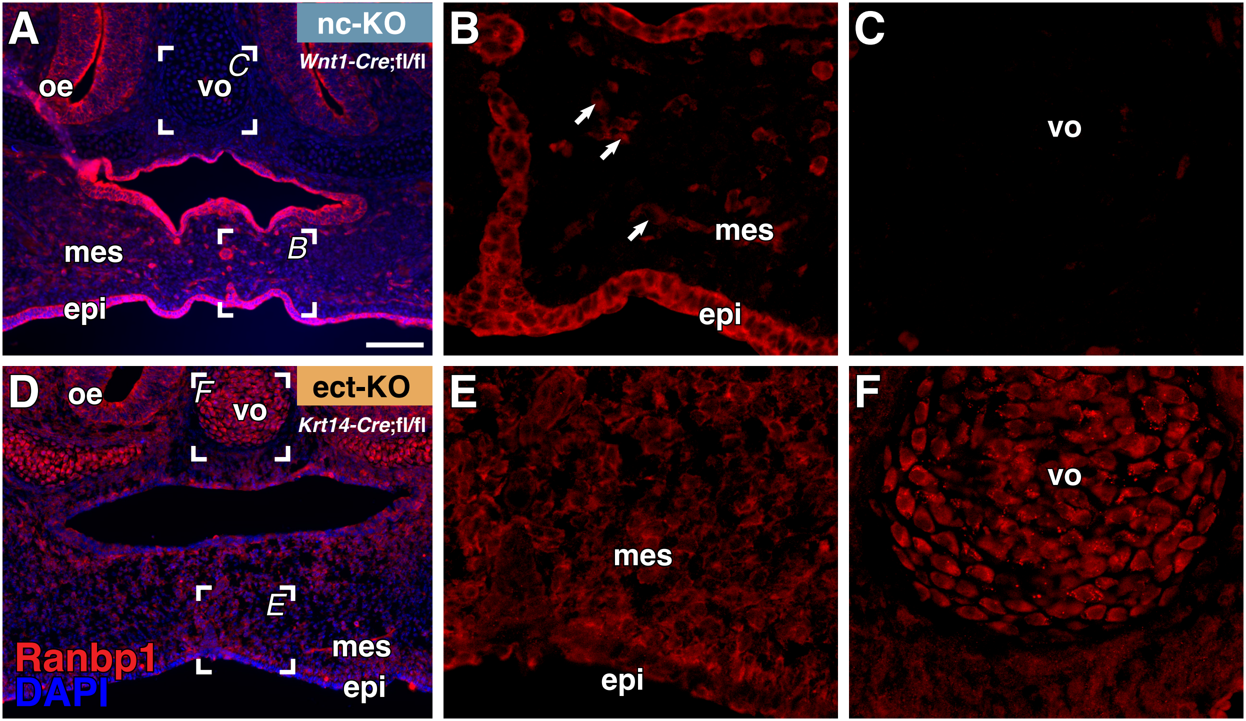

Supplemental Figure 5. Conditional knockout of Ranbp1 protein in developing orofacial structures. Sections of conditional knockouts at E15.5 were immunostained for Ranbp1 protein. (A-C) Conditional knockout of neural crest subpopulation (nc-KO) by Wnt1-Cre leads to a reduction of Ranbp1 expressing cells in crest-derived mesenchyme: widefield view at 20x magnification (A) illustrates loss of Ranbp1 expressing cells in craniofacial mesenchyme (mes), including in bone forming cells such as the vomer (vo), while epithelial (epi) expression remains robust. (B, C) 40x confocal images of adjacent sections to show loss of expressing cells in palate (B) and vomer (C). Some Ranbp1 expressing cells remain, particularly in the palate (B, arrows), reflecting either inefficiency of the Wnt1-Cre driver or diversity of the craniofacial mesenchymal population (e.g possible contribution of mesodermally-derived mesenchymal cells, vascular precursors, etc.). (D-E) Conditional knockout in the ectoderm (ect-KO) using the Krt14-Cre transgene likewise removes Ranbp1 protein expression from epithelial cells. Confocal views demonstrate robust expression in non-targeted palatal mesenchyme (mes, E) and cranial bone (vo, F).
