# Supplementary material for: Ranbp1 modulates morphogenesis of the craniofacial midline in mouse models of 22q11.2 deletion syndrome
Source: Hum Mol Genet. 2023 Feb 15;32(12):1959–74. doi: 10.1093/hmg/ddad030 (PMC10244217; doi:10.1093/hmg/ddad030)
Supplement: Ranbp1_Supplemental_Figures_6_ddad030 [file ranbp1_supplemental_figures_6_ddad030.pdf]

Supplemental Figure 6

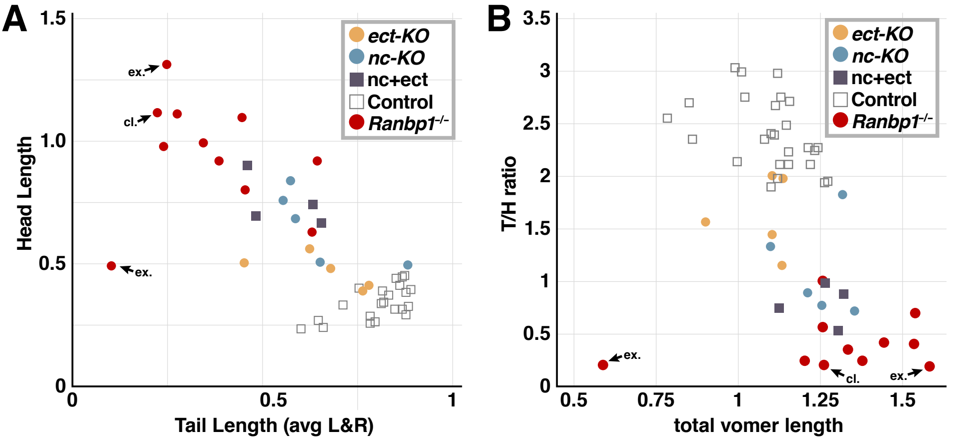

Supplemental Figure 6. Alternate visualizations of vomer morphology (as in Supp. Fig. 3) demonstrate overlap between conditional *Ranbp1* and constitutive *Ranbp1*$^{-/-}$ specimens. Conditional mutant vomers appear distinct from control specimens (combined WT and Cre-only); in contrast there is robust overlap between the nc-KO and nc+ect KO vomer morphology and the constitutive *Ranbp1*$^{-/-}$ samples.
