# Supplementary material for: Ranbp1 modulates morphogenesis of the craniofacial midline in mouse models of 22q11.2 deletion syndrome
Source: Hum Mol Genet. 2023 Feb 15;32(12):1959–74. doi: 10.1093/hmg/ddad030 (PMC10244217; doi:10.1093/hmg/ddad030)
Supplement: Ranbp1_Supplemental_Figures_7_ddad030 [file ranbp1_supplemental_figures_7_ddad030.pdf]

Supplemental Figure 7

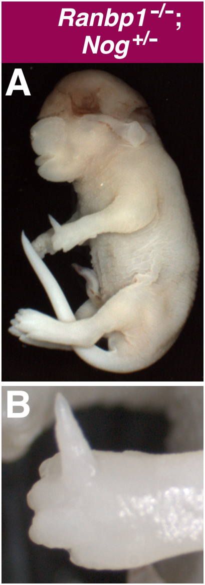

Supplemental Figure 7. Additional phenotype (polydactyly) in compound mutant. (A-B) One embryo with dorsal polydactyly was identified in the cohort of 4 *Ranbp1*$^{-/-}$;*Nog*$^{+/-}$ compound mutants. (B) The extra digit can be seen extending from the dorsal surface of the forefoot, between the second and third digits. This phenotype has not been described in either the *Ranbp1* nor *Nog* null mutants. Dorsal polydactyly is extremely rare and not well described in the literature, nor associated with any identified genetic syndrome.
