# Supplementary material for: Ranbp1 modulates morphogenesis of the craniofacial midline in mouse models of 22q11.2 deletion syndrome
Source: Hum Mol Genet. 2023 Feb 15;32(12):1959–74. doi: 10.1093/hmg/ddad030 (PMC10244217; doi:10.1093/hmg/ddad030)
Supplement: Ranbp1_Supplemental_Figures_8_ddad030 [file ranbp1_supplemental_figures_8_ddad030.pdf]

Supplemental Figure 8

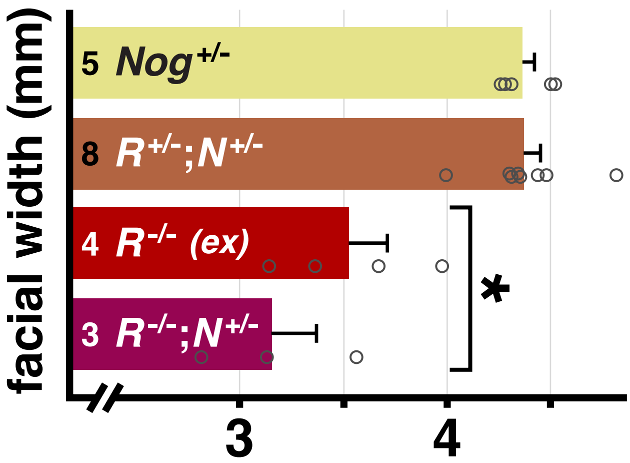

Supplemental Figure 8. Facial width measurements of *Ranbp1-Noggin* compound mutants, as measured in Fig. 1E-F. Compound *Ranbp1*$^{-/-}$;*Nog*$^{+/-}$ mutant facial measurements are narrower than in *Ranbp1*$^{-/-}$ embryos (P=0.04 by 1-way ANOVA with Šídák's multiple comparisons test).
