# Supplementary material for: Ranbp1 modulates morphogenesis of the craniofacial midline in mouse models of 22q11.2 deletion syndrome
Source: Hum Mol Genet. 2023 Feb 15;32(12):1959–74. doi: 10.1093/hmg/ddad030 (PMC10244217; doi:10.1093/hmg/ddad030)
Supplement: Ranbp1_Supplemental_Figures_9_ddad030 [file ranbp1_supplemental_figures_9_ddad030.pdf]

Supplemental Figure 9

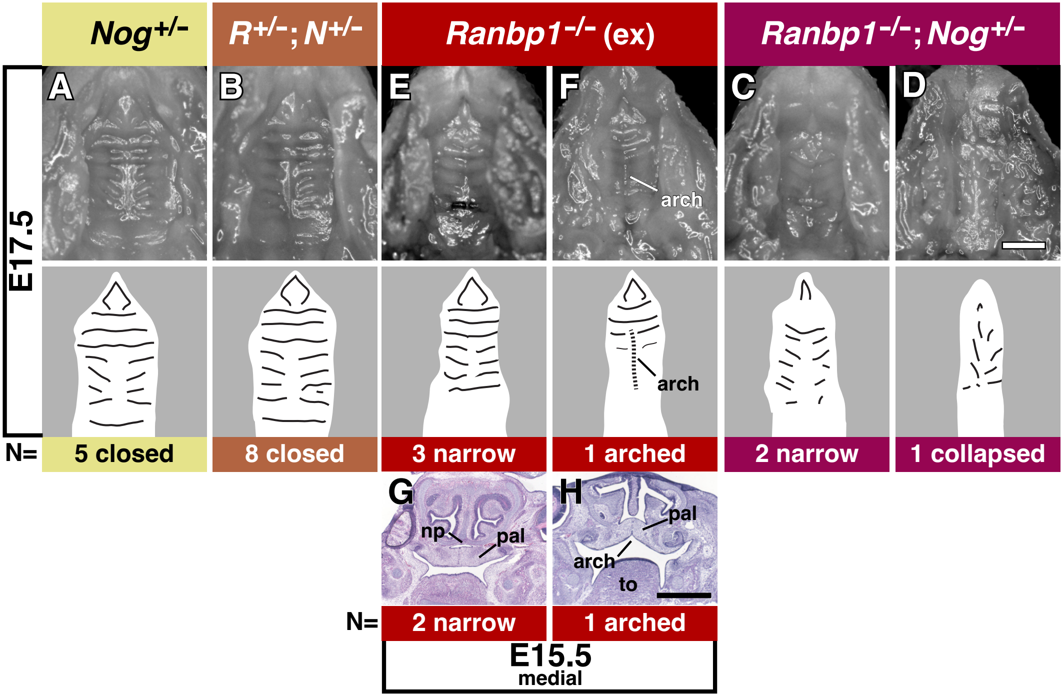

Supplemental Figure 9. Palate morphology of *Ranbp1-Noggin* compound mutants. (**A-F**) View of dissected palates from E17 embryos in *Ranbp1*;*Nog* allelic series; a line drawing of palatal features is presented below each for additional clarity. *Nog*$^{+/-}$ (**A**) and *Ranbp1*$^{+/-}$;*Nog*$^{+/-}$ (**B**) palates appear similar to WT, while exencephalic *Ranbp1*$^{+/-}$ palates are significantly narrower (**C-D**). Within the exencephalic *Ranbp1*$^{+/-}$ cohort, a subset have a more severe "arched" phenotype (**D**), presenting as a crease along the palatal midline, which is particularly evident in section (**H**, as compared to non-arched palate in **G**). (**E-F**) *Ranbp1*$^{-/-}$;*Nog*$^{+/-}$ palates show severe dysmorphology. In the most severe case (**F**), the palate is extremely narrow, with apparently disorganized palatal rugae. Scale bar = 1mm.
