# Supplementary material for: Ranbp1 modulates morphogenesis of the craniofacial midline in mouse models of 22q11.2 deletion syndrome
Source: Hum Mol Genet. 2023 Feb 15;32(12):1959–74. doi: 10.1093/hmg/ddad030 (PMC10244217; doi:10.1093/hmg/ddad030)
Supplement: Ranbp1_Supplemental_Figures_10_ddad030 [file ranbp1_supplemental_figures_10_ddad030.pdf]

Supplemental Figure 10

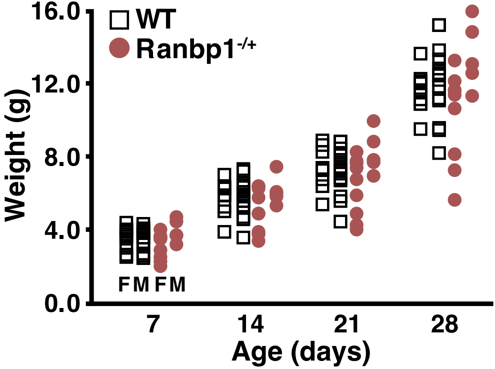

Supplemental Figure 10. Postnatal weight gain is not impaired in *Ranbp1*$^{+/-}$ pups. Litters of pups from a *Ranbp1*$^{+/-}$ x WT mating (wild-type mother) were weighed weekly from P7-P28 (WT: n=15 males, 10 females; *Ranbp1*$^{+/-}$ n=6 male, 11 female). No significant difference was seen in weight gain at P28 by genotype (P>0.6 by 2-way ANOVA, genotype x sex).
