# Supplementary material for: Ranbp1 modulates morphogenesis of the craniofacial midline in mouse models of 22q11.2 deletion syndrome
Source: Hum Mol Genet. 2023 Feb 15;32(12):1959–74. doi: 10.1093/hmg/ddad030 (PMC10244217; doi:10.1093/hmg/ddad030)
Supplement: Ranbp1_Supplemental_Figures_11_ddad030 [file ranbp1_supplemental_figures_11_ddad030.pdf]

Supplemental Figure 11

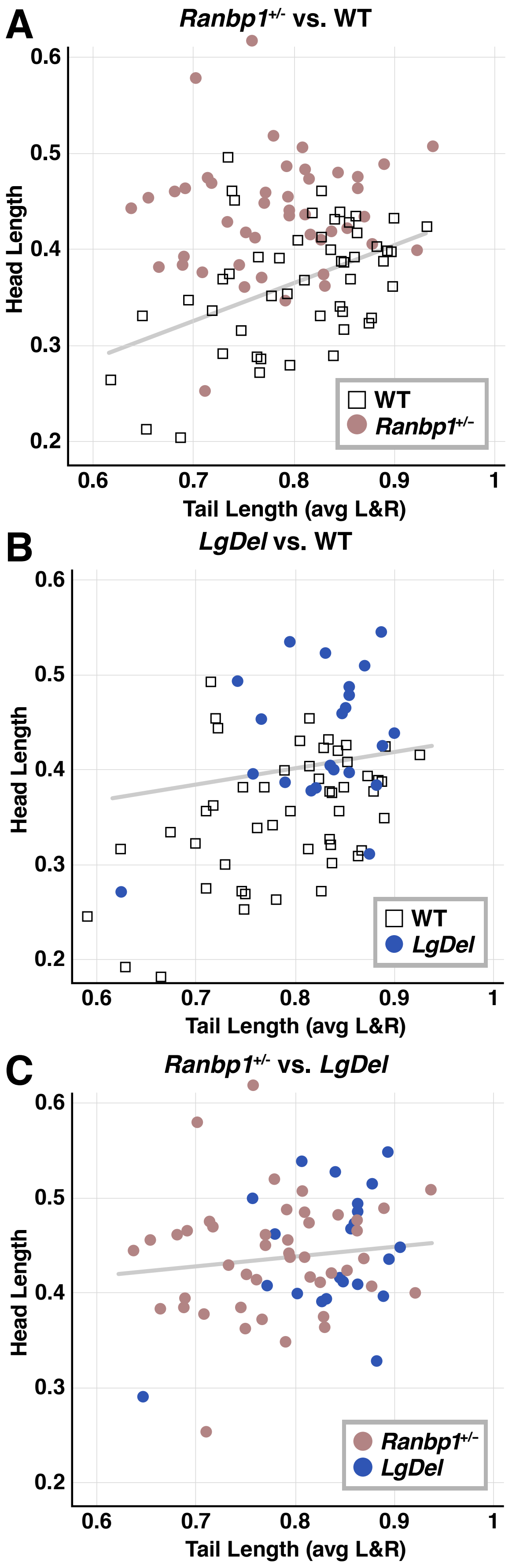

Supplemental Figure 11. Alternate analysis of vomer morphology for *Ranbp1*$^{+/-}$ and *LgDel* embryos. (A) To more clearly illustrate how WT and *Ranbp1*$^{+/-}$ vomers have distinct (although overlapping) morphologies, the same data from Fig. 8A was re-plotted by head length vs. tail length on an x-y scatter plot, rather than by the single dimension of the tail/head ratio. Further quantification was performed by producing a best-fit line to the WT dataset, and determining which points lie above or below the line, which confirmed that the two datasets are distinct (WT: 24 above, 26 below, *Ranbp1*$^{+/-}$: 5 above, 40 below; P<0.0001 by Fisher's Exact test). (B) A similar analysis for *LgDel* vomers using the same criteria also shows a clear distinction between genotypes, both graphically and statistically (*LgDel*: 2 above, 20 below; P<0.002 by Fisher's Exact test). (C) When *Ranbp1*$^{+/-}$ and *LgDel* are compared directly (using a best-fit line to the *Ranbp1*$^{+/-}$ dataset), the populations are indistinguishable (*Ranbp1*$^{+/-}$: 23 above, 22 below; *LgDel*: 12 above, 10 below; P>0.99 by Fisher's Exact test).
